# Antigen delivery to dendritic cells shapes human CD4^+^ and CD8^+^ T cell memory responses to *Staphylococcus aureus*

**DOI:** 10.1371/journal.ppat.1006387

**Published:** 2017-05-25

**Authors:** Julia Uebele, Christoph Stein, Minh-Thu Nguyen, Anja Schneider, Franziska Kleinert, Olga Tichá, Gabriele Bierbaum, Friedrich Götz, Isabelle Bekeredjian-Ding

**Affiliations:** 1 Division of Microbiology, Paul-Ehrlich Institute, Langen, Germany; 2 Institute for Medical Microbiology, Immunology and Parasitology (IMMIP), University Hospital Bonn, Bonn, Germany; 3 Interfaculty Institute of Microbiology and Infection Medicine (IMIT), University Tuebingen, Auf der Morgenstelle 28/E8, Tuebingen, Germany; Johns Hopkins School of Medicine, UNITED STATES

## Abstract

Intracellular persistence of *Staphylococcus aureus* favors bacterial spread and chronic infections. Here, we provide evidence for the existence of human CD4^+^ and CD8^+^ T cell memory against staphylococcal antigens. Notably, the latter could provide a missing link in our understanding of immune control of intracellular *S*. *aureus*. The analyses showed that pulsing of monocyte-derived dendritic cells (MoDC) with native staphylococcal protein antigens induced release of Th2-associated cytokines and mediators linked to T regulatory cell development (G-CSF, IL-2 and IL-10) from both CD4^+^ and CD8^+^ T cells, thus revealing a state of tolerance predominantly arising from preformed memory T cells. Furthermore, G-CSF was identified as a suppressor of CD8^+^ T cell-derived IFNγ secretion, thus confirming a tolerogenic role of this cytokine in the regulation of T cell responses to *S*. *aureus*. Nevertheless, delivery of *in vitro* transcribed mRNA-encoded staphylococcal antigens triggered Th1-biased responses, e.g. IFNγ and TNF release from both naïve and memory T cells. Collectively, our data highlight the potential of mRNA-adjuvanted antigen presentation to enable inflammatory responses, thus overriding the existing Th2/Treg-biased memory T cell response to native *S*. *aureus* antigens.

## Author summary

*Staphylococcus aureus* is deemed one of the most important nosocomial pathogens but, to date, there are no safe and protective vaccines. In this study we investigate the nature of the preformed T cell response to *S*. *aureus* antigens in healthy donors. Our data reveal that CD4^+^ and—so far not described—CD8^+^ T cell memory responses against native staphylococcal antigens exist but are skewed towards minimizing inflammation and promoting tolerance. The T cell response to staphylococcal antigens is characterized by the secretion of typical Th2 cytokines such as IL-5 and IL-13 and mediators associated with formation of T regulatory cells. Most importantly, G-CSF suppresses IFNγ release from pre-existent memory T cells. However, our data reveal that the use of mRNA-encoded antigens to trigger *S*. *aureus*-specific T cell responses bears the potential to override the tolerogenic bias. It favors TNF- and IFNγ-releasing T cells and may, thus, represent an innovative tool in prophylactic and therapeutic vaccine development.

## Introduction

*Staphylococcus aureus* colonizes the skin and mucosa of approximately 50–60% of adults independent of their country and origin. Chronic exposure to this pathogen increases risk of infection [1]. However, infection occurs despite the presence of antigen-specific antibodies against a variety of staphylococcal antigens and *S*. *aureus* is among the most common causes of nosocomial infections worldwide [2]. Furthermore, all clinical vaccine trials issued to date have failed to demonstrate protection despite promising results in preclinical mouse models [3–6], which emphasizes the need to define a human correlate of protection in infections with commensals.

Recent studies highlighted the importance of T cells, in particular Th1 and Th17 cells, for bacterial clearance in murine models for nasal colonization and cutaneous infection [7–14]. In the human host, however, only scarce information is available on T cell immunity against *S*. *aureus*. Only recent studies have addressed this question and provide evidence for the existence of an anti-staphylococcal T cell repertoire in healthy donors and patients [15,16].

Notably, one of these studies estimated that in healthy individuals T cells specific for extracellular staphylococcal antigens account for 0.2 to 5.7% of total peripheral blood T cells [16]. Furthermore, CD4^+^ memory T cell responses were described in patients with blood stream infections and, in mice, adoptive transfer or induction of antigen-specific Th1 responses was protective against invasive infection [15]. Nevertheless, the exact regulation of the T cell response against *S*. *aureus* remains largely unexplored.

It has been proposed that intracellular persistence facilitates chronic colonization of the respiratory compartment [17,18]. Reasoning that CD4^+^ T helper cells and antibodies are not effective against intracellularly residing bacteria we sought to investigate whether there is a preformed CD8^+^ T cell response to *S*. *aureus* in humans. These cells have the ability to recognize intracellularly processed bacterial antigens via MHC class I and to kill infected cells. They could, therefore, serve as important sentinels in controlling *S*. *aureus* at the human mucosal surfaces.

To detect CD8^+^ T cell responses we used *in vitro* transcribed (ivT) mRNA for antigen delivery, a method well-established in tumor immune therapy for the induction of cytotoxic T cell responses against cells presenting tumor antigens via MHCI [19–23]. Recently, this technique was also exploited to vaccinate against viral infection [24–26]. Thus, we hypothesized that this approach could represent an effective tool for the induction of CD8^+^ T cell responses against an intracellularly persisting bacterial pathogen. In particular, the mechanisms enabling immune tolerance to commensal pathogens such as *S*. *aureus* might resemble those present in a tumor environment. With this technique we provide evidence for the presence of CD8^+^ T cells specific for *S*. *aureus* antigens in human healthy donors. Furthermore, comparison of ivT mRNA and protein-mediated antigen presentation confirms our hypothesis by demonstrating the tolerogenic properties of the established human CD4^+^ and CD8^+^ T cell memory response to native *S*. *aureus* proteins.

## Results

### Dendritic cells loaded with mRNA encoding *S*. *aureus* antigens trigger interferon-γ (IFNγ) secretion in human CD8^+^ T cells

The major objective of this study was to analyze CD8^+^ T cell responses against staphylococcal antigens. To this end, we cloned three different genes encoding important staphylococcal antigens into the pT7CFE1-cMyc vector for *in vitro* transcription: 1. *sitC*, which encodes a major lipoprotein involved in iron transport often used as model TLR2 (Toll-like receptor 2) agonist [27,28], 2. *spa*, which encodes staphylococcal surface protein A (SpA), a core variable gene [29], and 3. *mecA*, an accessory gene encoding the penicillin binding protein 2a (PBP2a), which confers methicillin resistance [30].

For T cell activation mRNAs encoding *spa*, *mecA* or *sitC* were transfected into MoDC and co-cultured with autologous CD8^+^ or CD4^+^ T cells. IFNγ-secreting cells were visualized by ELISPOT after overnight incubation (S1A Fig). Unspecific T cell activation was visualized using non-coding mRNA from the backbone of the original pT7CFE1-cMyc vector, subsequently referred to as background control (non-coding, NC) (S1A Fig). Lipofection (LF) alone did not induce IFNγ production (S1A Fig). As expected due to the method inherent predominance of MHCI-restricted peptide presentation [21] the number of IFNγ-secreting cells was higher in the CD8^+^ than in the CD4^+^ T cell fraction (S1A Fig). Furthermore, the number of IFNγ-secreting T cells could be enriched by isolating CD45RO^+^ T cells, indicating that IFNγ is released from pre-existent memory T cells (S1B Fig).

### mRNA-encoded antigen supersedes protein antigen in the induction of IFNγ production

**Fig 1:**
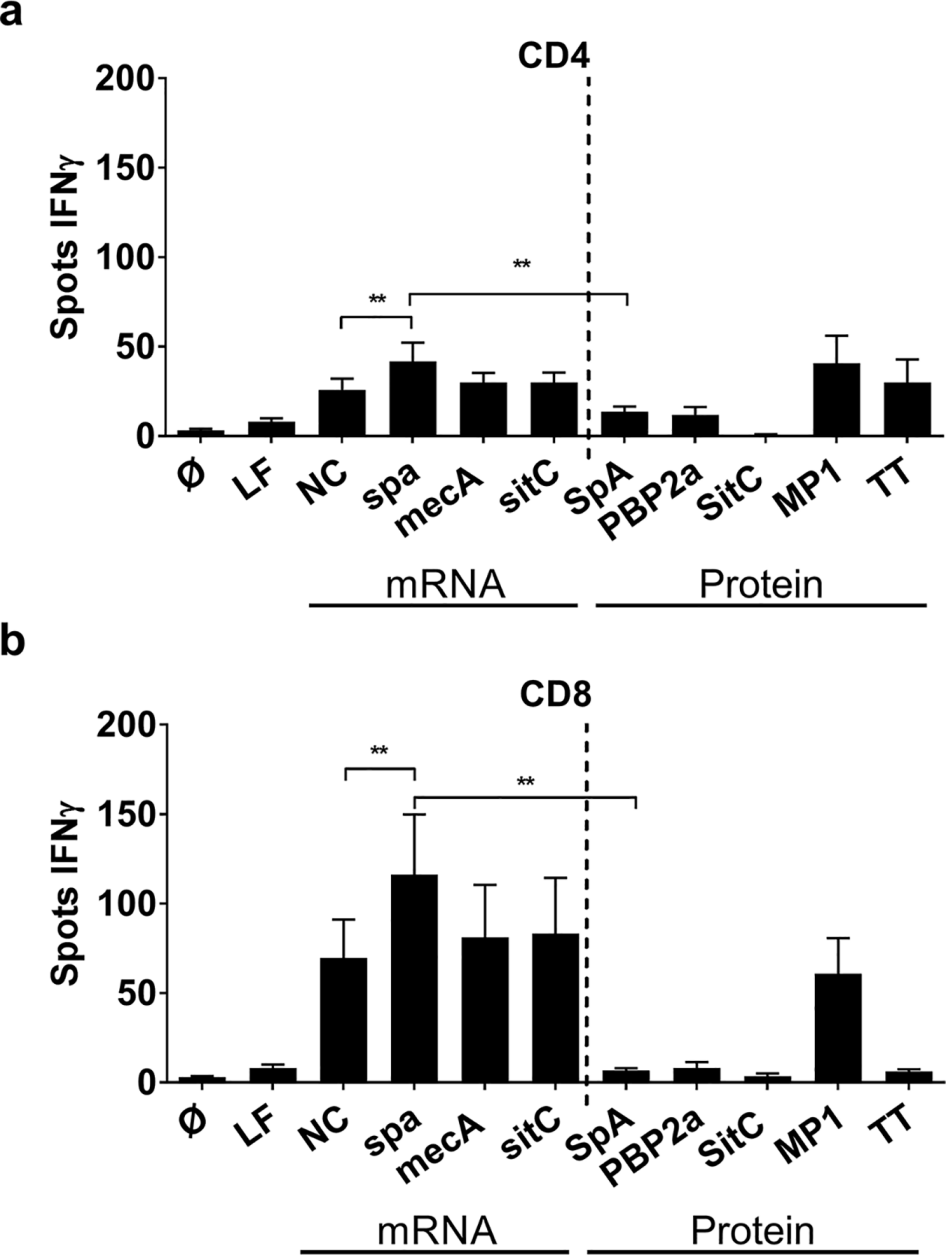
Comparison of IFNγ production by T cells stimulated with mRNA- or protein-derived staphylococcal antigens. **(a)** CD4^+^ T cells and **(b)** CD8^+^ T cells in co-culture with MoDC were stimulated with either mRNA-encoded antigens or the corresponding protein antigens, e.g. *spa* / SpA, *mecA* / PBP2a and *sitC* / SitC. Lipofectamine (LF) alone, non-coding mRNA (NC) and a peptide pool from matrix protein 1 (MP1) of H1N1 Influenza virus and Tetanus toxoid (TT) served as controls. The number of IFNγ ELISpot spots after overnight culture is shown as mean values ± SEM of n = 8 donors. p**<0.01, p*< 0.05 (Wilcoxon matched-pairs signed rank test). Experiments were done in duplicates.

To exclude immune effects owing to the ivT mRNA technique used for antigen presentation we, next, compared the T cell stimulatory capacity of mRNA-encoding antigens to that of the corresponding protein antigens. To this end, we delivered antigen to MoDC, co-incubated these with CD4^+^ or CD8^+^ T cell fractions and quantified IFNγ secretion by ELISPOT. Tetanus toxoid (TT) and a peptide pool from the Influenza H1N1 matrix protein 1 (MP1) were used as recall antigen controls for CD4^+^ and CD8^+^ T cell activation, respectively. Independent of the antigen, IFNγ secretion was up to three-fold higher in CD8^+^ T cells than in CD4^+^ T cells (Fig 1). However, of the three antigens delivered via mRNA, only *spa-*encoding mRNA significantly exceeded induction of IFNγ by non-coding mRNA in both CD4^+^ and CD8^+^ T cells. Most importantly, protein antigens failed to induce IFNγ to the extent achieved by stimulation with mRNA-encoded antigens in both T cell fractions (Fig 1). By contrast, MP1 peptides induced strong IFNγ release in CD8^+^ and CD4^+^ T cells and, as expected, TT predominantly stimulated CD4^+^ T cells (Fig 1) [31].

### Intrinsic immune stimulatory properties of mRNA facilitate IFNγ induction

**Fig 2:**
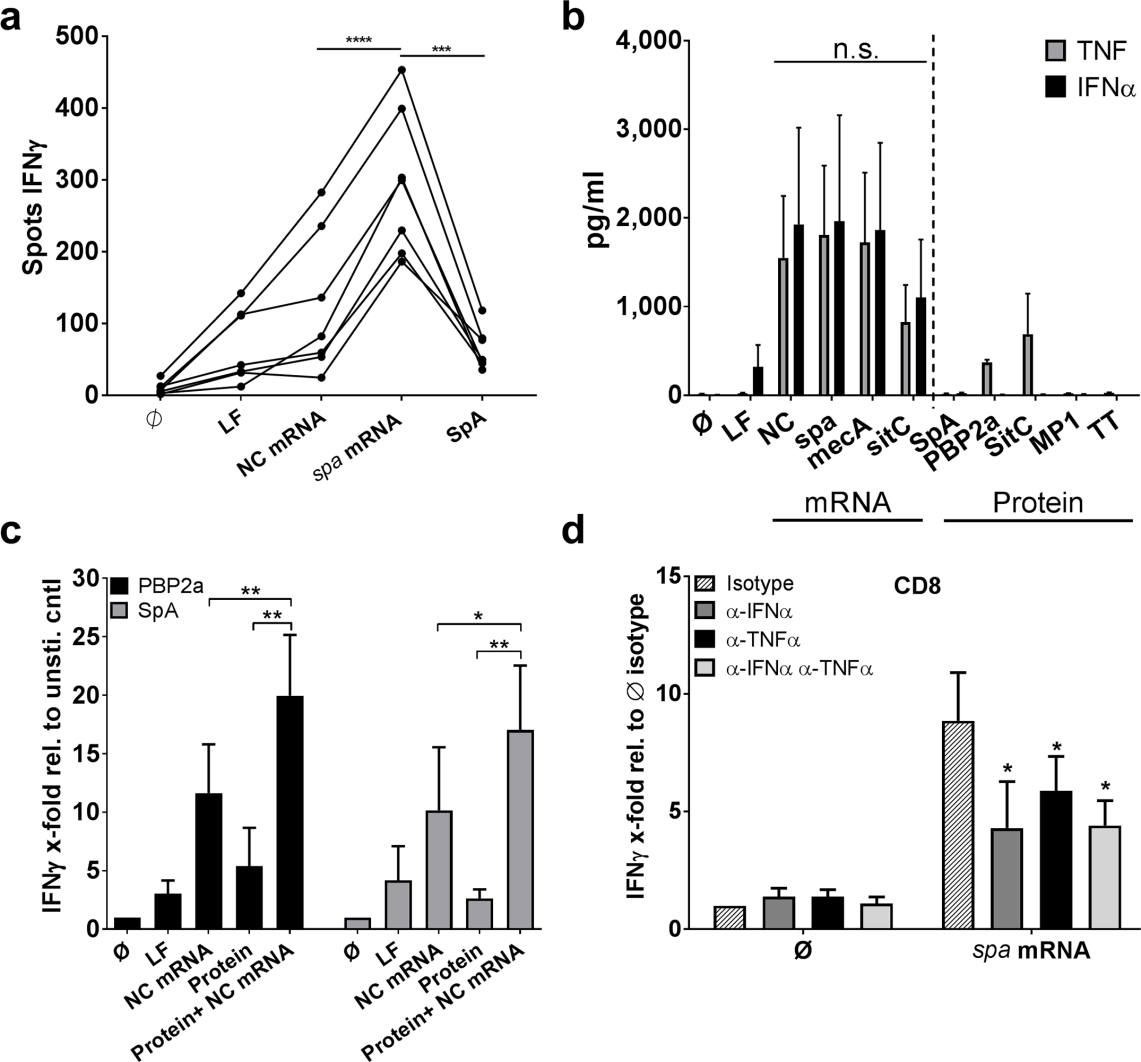
mRNA-promoted cytokine response and adjuvant effect. **(a)** PBMC were co-cultured with autologous MoDC loaded with either non-coding control mRNA (NC), *spa* mRNA, SpA protein or the lipofection control (LF). IFNγ was detected after overnight incubation by ELISpot. The individual results of the single donors (n = 7) were connected with lines and displayed as IFNγ spots. Analysis was carried out in technical duplicates. Following testing for normal distribution, paired Student’s t-test was used to calculate significance. **(b)** Cytokine production by MoDC stimulated with mRNA-encoded staphylococcal antigens or the respective proteins and controls (lipofectamine (LF), non-coding control mRNA (NC), Influenza MP1 peptides (MP1) and tetanus toxoid (TT)) was measured in supernatants after 24 hours. The results of duplicates for TNF and IFNα are shown as mean values ± SEM of n = 4 donors. p≥ 0.05 n.s. (One-way ANOVA) **(c)** Stimulation of MoDC/CD8^+^ T cell co-cultures with PBP2a or SpA in the presence and absence of NC mRNA after overnight IFNγ ELISPOT analysis. The ELISPOT enzymatic activity relative to the unstimulated control is shown as mean values ± SEM of n = 8 independent donors. Statistical analysis was done using the Wilcoxon matched-pairs signed rank test. Experiments were carried out in duplicates. **(d)** Upon transfection of MoDC with *spa* mRNA and co-culture with CD8^+^ T cells, blocking antibodies against IFNα and TNFα alone or in combination were added and IFNγ secretion of duplicates was quantified by ELISPOT. IFNγ enzymatic activity of n = 5 independent donors is normalized to the unstimulated isotype control and displayed as mean ± SEM. Following testing for normal distribution, paired Student’s t-test was used to calculate significance. p**<0.01, p*< 0.05.

In contrast to proteins or peptides, intracellular delivery of ivT mRNA is known to stimulate RNA-sensing pattern recognition receptors (PRR) such as TLR8 and RIG-I [32,33]. To assess the impact of mRNA-mediated PRR activation in a more physiological setting we analyzed IFNγ production in PBMC in co-culture with MoDC loaded with SpA protein and non-coding or *spa*- encoding mRNA. ELISpot analysis demonstrated significantly stronger IFNγ production in the presence of *spa* mRNA when compared to the background control and SpA stimulation after overnight incubation (Fig 2A). Next, we loaded MoDC with mRNA or protein antigens. The results showed that lipofection of ivT mRNA induced high levels of tumor necrosis factor (TNF) and IFNα independent of the encoded antigen (Fig 2B). By contrast, when MoDC were stimulated with TLR2-active SitC lipoprotein TNF reached similar levels but IFNα was not detectable. Moreover, recombinant PBP2a and *S*. *aureus*-derived SpA induced very little or none of these cytokines, respectively. Of note, low levels of IL-1β and IL-10 were detected in all conditions with exception of influenza MP1 peptides and tetanus toxoid (S2 Fig).

Subsequently, we combined *S*. *aureus* protein antigens with non-coding ivT mRNA (NC). In the presence of ivT mRNA IFNγ responses to PBP2a and SpA reached significant higher levels than NC mRNA alone (Fig 2C). Finally, blocking of IFNα and TNF with neutralizing antibodies showed that absence of MoDC-derived IFNα decreased T cell-mediated IFNγ release by approximately 50% while neutralization of TNF was less potent and had no additional effect when both cytokines were blocked (Fig 2D). IFNγ responses to *S*. *aureus* antigens are, thus, enhanced by MoDC-derived cytokines and differences in the number and composition of innate immune cells and the respective cytokine secretion can strongly affect the T cell response.

### The endogenous T cell response to *S*. *aureus* proteins is dominated by Th2-associated cytokines

**Fig 3:**
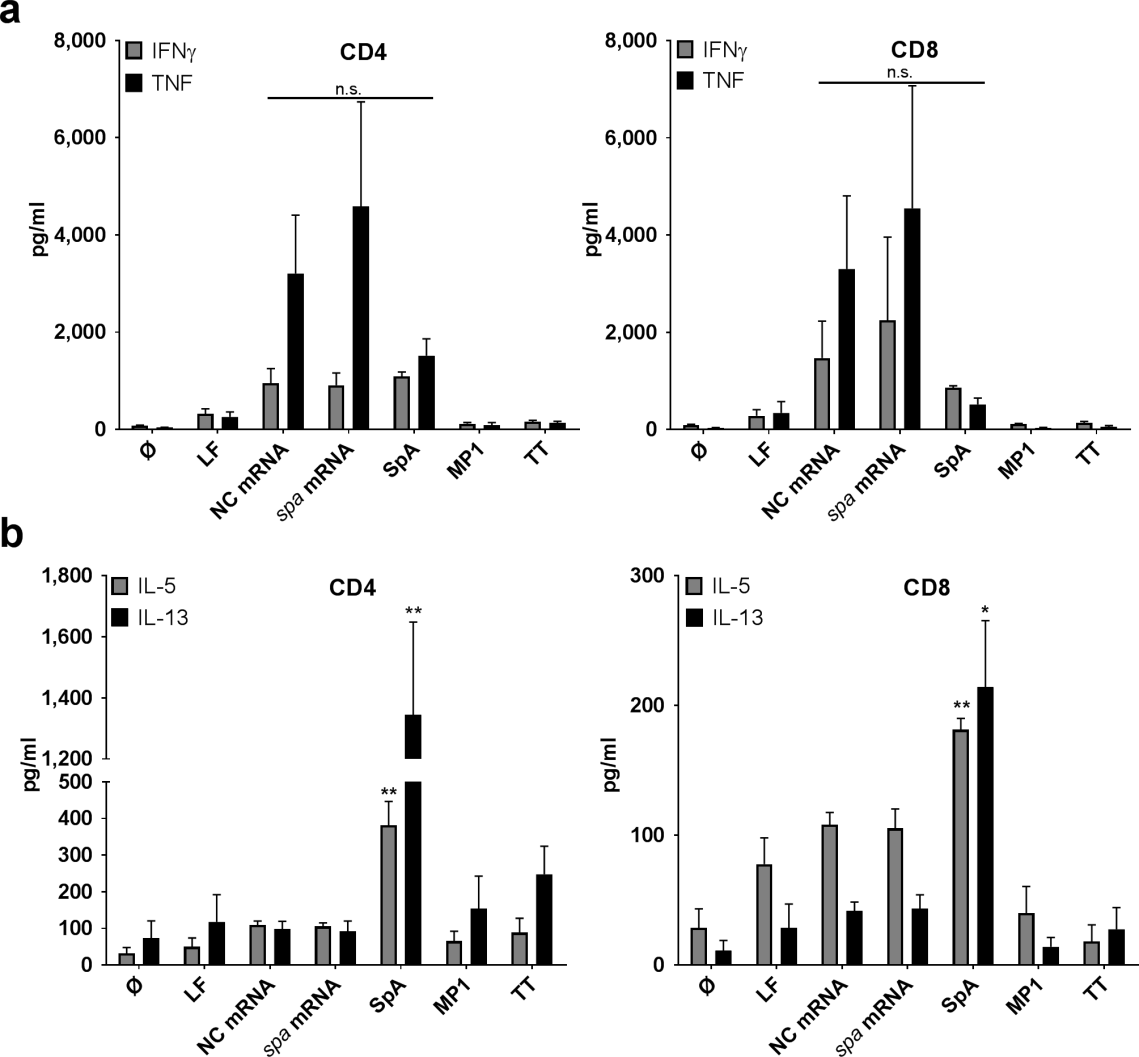
T cell cytokine profiles in response to *spa* mRNA-encoded and SpA protein delivered antigens. Cytokine secretion profiles in day 5 supernatants of CD4^+^ (left panel) or CD8^+^ T cells (right panel) stimulated with *spa* mRNA or SpA protein antigens was performed using a multiplex cytokine array: **(a)** Th1 cytokines (IFNγ, TNF) and **(b)** Th2 cytokines (IL-5, IL-13). The graphs depict the mean values ± SEM obtained from n = 6 independent donors. Experiments were carried out in duplicates. For Th1 cytokines, one-way ANOVA was used to test significance of multiple conditions; for Th2 cytokines, p values refer to condition with *spa* mRNA (paired student’s t-test; p**<0.01, p*< 0.05).

To further characterize the T cell response against *S*. *aureus*, we analyzed T cell-derived cytokine secretion patterns in 5 day co-cultures with MoDC transfected with *spa* mRNA or SpA protein pulsed MoDC. Analysis of IFNγ secretion in supernatants from CD8^+^ T cells confirmed the results obtained by ELISPOT with highest levels in conditions with MoDC transfected with mRNA-encoded SpA (Fig 3A). However, in the supernatants of CD4^+^ T cells IFNγ induced by SpA protein reached the levels induced by MoDC transfected with mRNA-encoded *spa*. However, this time frame (5 days) allows priming of naïve T cells, which cannot be achieved after overnight incubation for IFNγ ELISpot [34]. Notably, non-coding ivT mRNA induced high background levels of TNF in both T cell fractions but levels obtained with *spa*-encoding ivT mRNA were higher while only low amounts of TNF were detectable with SpA protein (Fig 3A). Of note, the total amount of TNF in MoDC/T cell co-cultures was markedly higher than in supernatants of MoDC stimulated with mRNA. Since co-cultures contained only 20.000 MoDC/well (Fig 3A) versus 100.000 MoDC/well in Fig 2B this indicates that T cells are the primary source of TNF.

Contrary to the results obtained for Th1- associated cytokines, pulsing of MoDC with SpA protein elicited significantly higher production of IL-5 and IL-13 from T cells, which was nearly absent in conditions with mRNA-based antigen delivery and in CD8^+^ T cells (Fig 3B). Notably, similar results were obtained for comparisons of *mecA*/PBP2a in CD4^+^ and CD8^+^ T cells (S3 Fig). These cytokines were nearly absent in both T cell fractions when MoDC were loaded with SitC (S3A Fig).

Furthermore, IL-4, one of the hallmarks of a Th2 response, was unspecifically triggered in the presence of mRNA (S3B Fig). Similarly, IL-17, which was reported to play an important role in *S*. *aureus* clearance [35,8,9,13], reached high levels induced by both mRNA-encoded antigens and proteins (PBP2a and SpA) in CD4^+^ and CD8^+^ T cell fractions but secretion induced by mRNA-encoded antigens did not exceed background levels (S3A Fig). Moreover, control antigens TT and MP1 peptides induced only low concentrations of IL-13 and IL-5 in CD8^+^ T cells and levels of IL-5 comparable to mRNA treated conditions in CD4^+^ T cells. Here, only low amounts of IL-17 were detected in both T cell fractions. Production of IL-8 and IL-6, important markers for innate immune cell activation and chemotaxis or cellular survival and growth, respectively, was detectable in all conditions (S4 Fig).

### Protein-derived antigens target memory T cell populations

**Fig 4:**
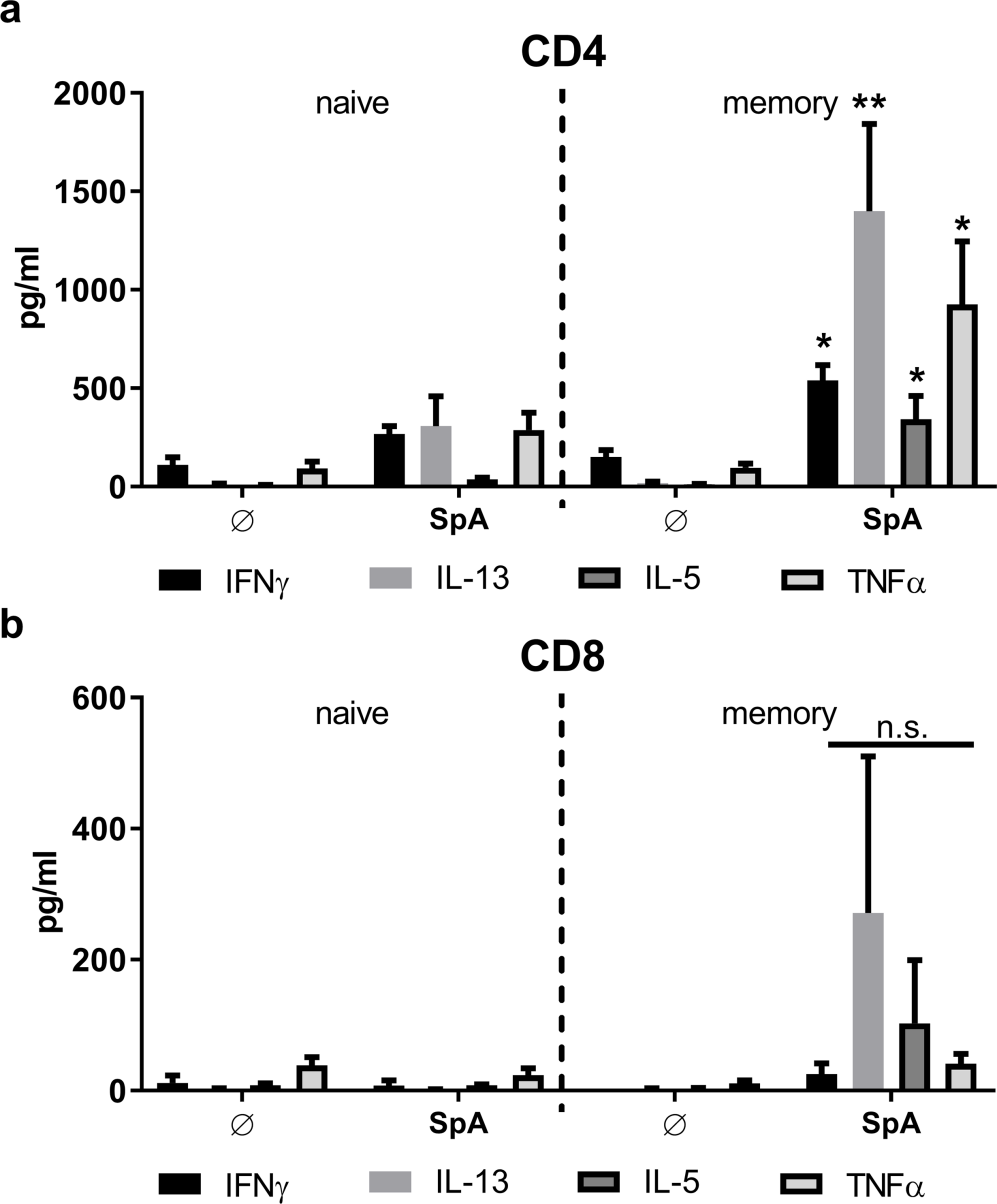
Protein-derived antigens activate memory T cells. Human **(a)** CD4^+^ or **(b)** CD8^+^ T cells were isolated from frozen PBMC via magnetic beads. Cell fractions were either purified for **(a)** CD14^-^CD8^-^ and **(b)** CD14^-^CD8^+^, and CD45RO^-^CD45RA^+^ (naïve) or CD45RO^+^CD45RA^-^ (memory) phenotype. Cytokine secretion profiles after 5 days of MoDC/T cell co-culture stimulated with SpA protein were measured by multiplex cytokine array, done in duplicates. TNF, IFNγ, IL-5 and IL-13 are presented as mean ± SEM of n = 7 or 8 donors, respectively. p value refers to the same condition in naïve T cells. p**<0.01, p*< 0.05 (paired student’s t-test). For testing of multiple conditions, one-way ANOVA was used.

To provide further proof that the T cell responses arise from a pre-existent T cell memory pool we sorted naïve (CD45RO^-^CD45RA^+^) and memory (CD45RO^+^CD45RA^-^) CD4^+^ and CD8^+^ T cells by flow cytometry (see Materials and Methods section, S8 Fig and S9 Fig) and stimulated these cells with MoDC transfected with *spa* mRNA or pulsed with SpA protein. As shown in Fig 4, SpA protein induced significantely higher levels of cytokines (IL-13 > TNF > IFNγ > IL-5) in the CD4^+^ memory T cell fraction (Fig 4A). And, despite not significant, an increase in IL-13, IL-5 and TNF derived from CD8^+^ memory T cells was also observed (Fig 4B). However, here, the total amount of cytokines was much lower because Th2-associated cytokines are characteristic of CD4^+^ T helper cells. By contrast, no differences in cytokine secretion levels (TNF >> IFNγ > IL-5 > IL-13) were observed between naïve and memory CD4^+^ T cells when antigen (*spa*) was provided as mRNA (S5 Fig).

### Th1 and Th2 responses to the same antigen arise from different T cell pools

**Fig 5:**
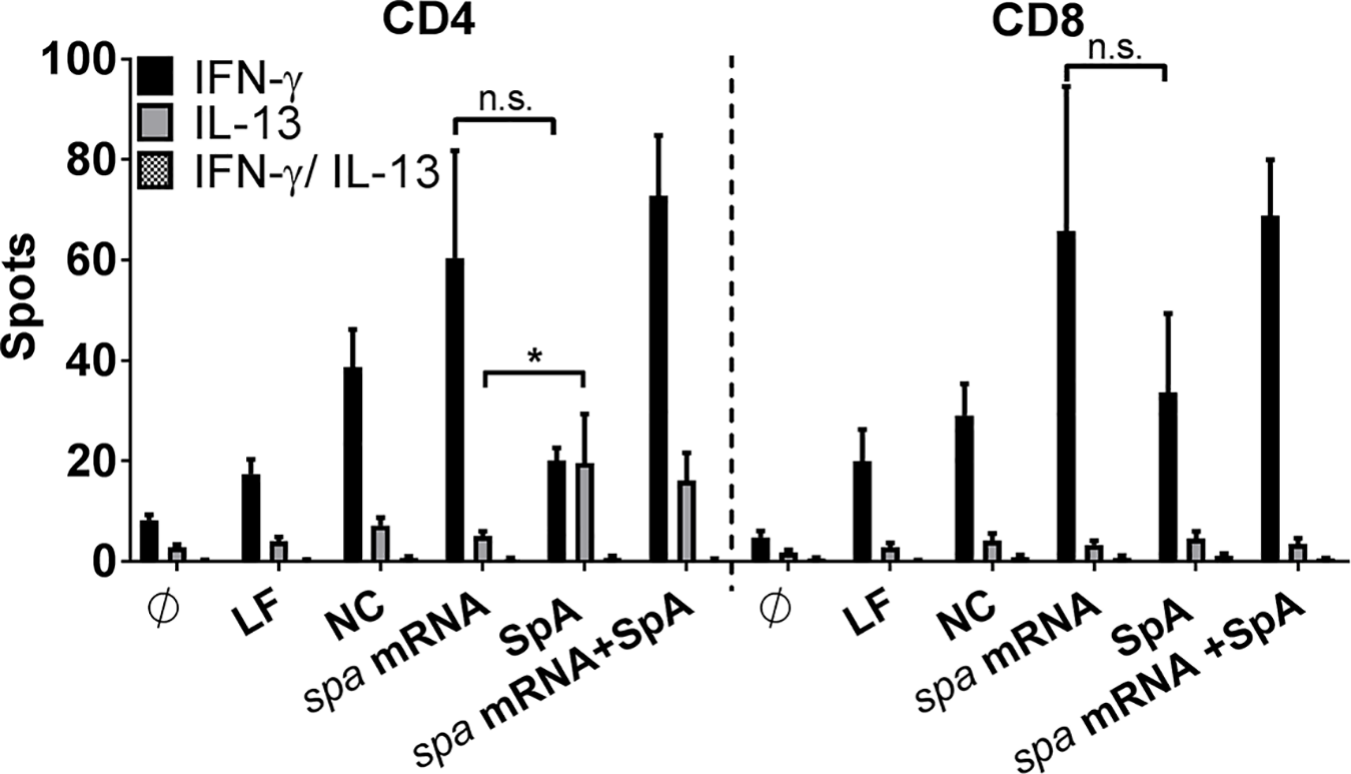
Antigen source-dependent activation of different T cell subsets. MoDC/T cell co-cultures of n = 8 independent donors were stimulated with *spa*-encoding mRNA and/or SpA protein and analyzed by IFNγ/IL-13 FluoroSpot demonstrating cytokine secretion by different cell subsets dependent on the antigen delivery. The number of spots is displayed as mean values ± SEM. p*< 0.05, n.s. not significant (Wilcoxon matched-pairs signed rank test). Experiments were done in technical duplicates.

Next, we hypothesized that addition of ivT mRNA and/or mRNA-encoded staphylococcal antigens could repolarize the preformed T cell response to native staphylococcal proteins. We, therefore, asked whether ivT mRNA and/or mRNA-encoded antigens activate a T cell pool identical to that found responsive to the corresponding protein antigen. To this end IFNγ/IL-13 FluoroSpot was performed after 5 days of MoDC/T cell co-culture. The results revealed that there was no overlap of IFNγ and IL-13 secretion from the same T cell source (Fig 5). They further showed that antigen presentation with SpA protein induced two non-overlapping subsets of CD4^+^ T cells that secreted IFNγ or IL-13, respectively. In the CD8^+^ T cell fraction presentation of SpA protein antigen induced IFNγ-secreting T cells but IL-13 was undetectable. Furthermore, MoDC stimulation with ivT mRNA encoding *spa* in parallel to pulsing with SpA protein did not abolish IL-13 secretion in CD4^+^ T cells but increased the amount of IFNγ-secreting T cells (Fig 5).

### Protein antigen-induced G-CSF interferes with IFNγ responses in CD8^+^ T cells

**Fig 6:**
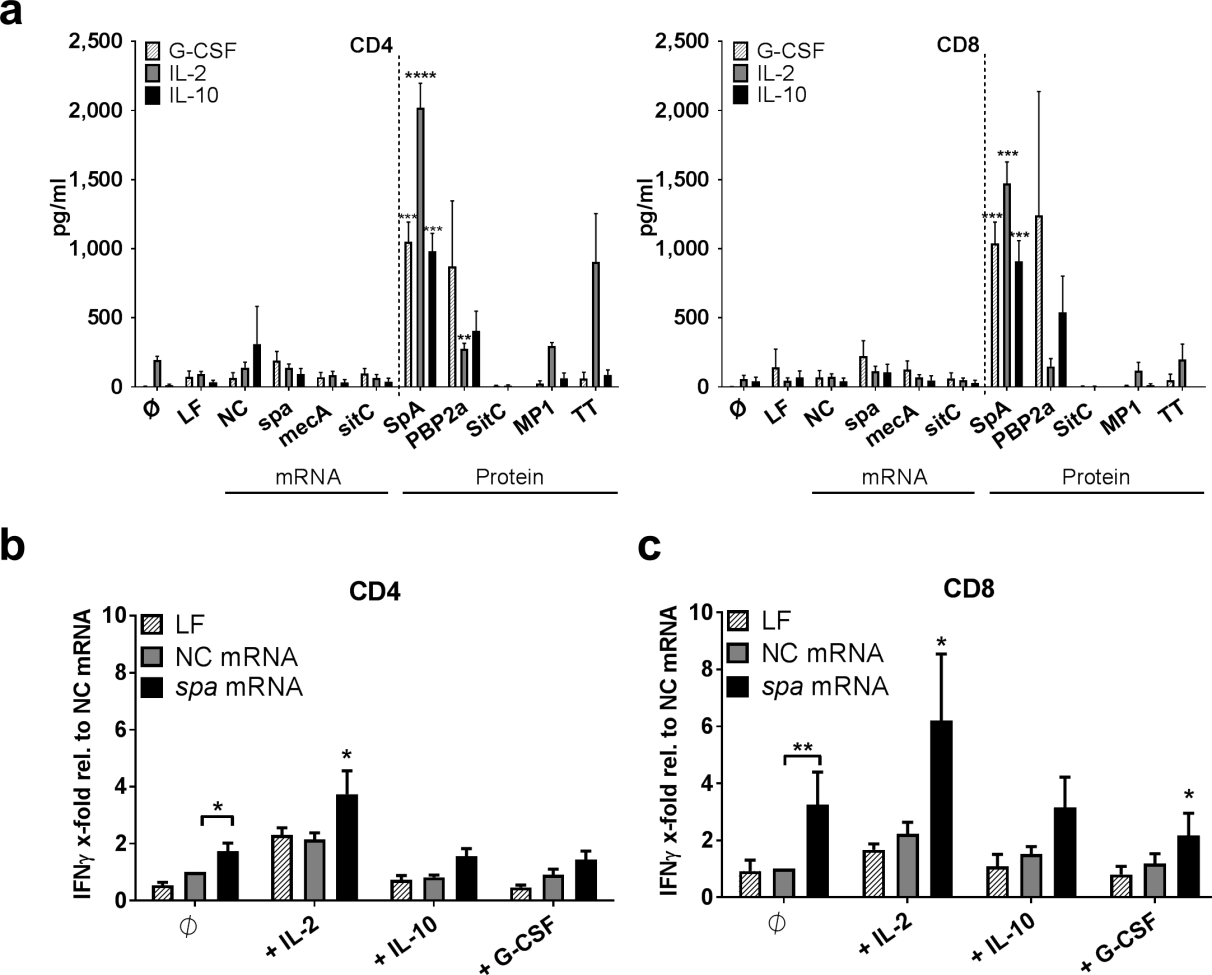
G-CSF-mediated regulation of IFNγ production. Cytokine secretion profiles of **(a)** G-CSF, IL-2 and IL-10 in day 5 supernatants of CD4^+^ (left panel) or CD8^+^ T cells (right panel): MoDC/T cell co-cultures stimulated with *spa*, *mecA or sitC* mRNA or the corresponding protein antigens (SpA, PBP2a, SitC) were performed using a multiplex cytokine array. The graphs depict the mean values ± SEM obtained by n = 6 independent donors. Paired t-test was used to test for significance. The p-value refers to the mRNA condition of the corresponding protein antigen and cytokine. **(b+c)** MoDC were transfected with non-coding (NC) or *spa* mRNA and cultured in the presence of recombinant IL-2, IL-10 or G-CSF with **(b)** CD4^+^ or **(c)** CD8^+^ T cells. IFNγ ELISPOT enzymatic activity of n = 8 different donors was normalized to the NC mRNA control. Wilcoxon matched-pairs signed rank test was used to test for significance. If not indicated otherwise, p values refer to the untreated control transfected with *spa* mRNA. p**<0.01, p*< 0.05. All experiments were done in duplicates.

Further analyses detected secretion of granulocyte-colony stimulating factor (G-CSF), IL-2 and IL-10 in CD4^+^ and CD8^+^ T cell fractions. Secretion of these cytokines was, however, restricted to conditions where MoDC were pulsed with *S*. *aureus* protein antigen, i.e. SpA and to a minor extent PBP2a (Fig 6A). Stimulation with SitC did not induce these mediators. Since suppressive properties of all of these cytokines have been described [36–39] this prompted us to ask whether release of these cytokines could be involved in the induction of immune tolerance to *S*. *aureus*. We, therefore, transfected MoDC with mRNA-encoded *spa* in the presence and absence of recombinant IL-2, IL-10 or G-CSF. The results showed that IL-2 enhanced *spa*-specific IFNγ responses in CD4^+^ (Fig 6B) and CD8^+^ T cells (Fig 6C), whereas IL-10 secretion did not affect *spa*-specific IFNγ release. However, addition of G-CSF interfered with IFNγ responses, in particular in *spa* mRNA-triggered CD8^+^ T cell responses (Fig 6C).

## Discussion

This study provides a state-of-the-art *ex vivo* analysis of T cell responses to *S*. *aureus* in healthy individuals, independent of their carrier state. The techniques used for these analyses have been optimized to enumerate T cell reactivity to tumor antigens in clinical immune monitoring in oncological trials using dendritic cell vaccines or other immune-based therapies. The use of a standardized protocol for the generation of MoDC minimizes donor-dependent differences in the antigen presenting compartment and, thus, allows standardization of antigen presentation and T cell stimulation and subsequently a better comparison of individual T cell responses. This is an important advantage when compared to limiting dilution assays performed with PBMC that display huge donor-dependent differences in the composition of antigen presenting leukocyte subpopulations [40,41] and were employed in earlier studies on *S*. *aureus*-specific T cells [16]. Similarly, the ELISPOT method is a well accepted and sensitive tool for *ex vivo* quantification of the frequency of antigen-specific memory T cells. It allows visualization of single T cells, predominantly T effector memory cells that are more reactogenic than naïve T cells [40,42]. The short time frame avoids *in vitro* amplification, an important confounder in classical assays such as chromium release assay and limiting dilution assays that require T cell expansion to increase sensitivity [40,41]. Notably, these methodological differences may account for differences in the quantitative and qualitative outcome of T cell responses to other studies that observed higher frequencies of antigen-specific T cells or predominant Th1 responses [16,43,15,44].

Considering its ability to persist intracellularly, *S*. *aureus*-specific CD8^+^ T cell responses might represent an important, so far unrecognized, cellular component in control of *S*. *aureus* spread and infection [45]. Despite increased susceptibility of IFNγR-deficient mice to *S*. *aureus* infections [46] the role of CD8^+^ T cells, classical IFNγ-producers, has neither been investigated nor have infection models addressed the T cell response to intracellularly persisting *S*. *aureus*. This study provides proof for the existence of a human CD4^+^ and, more important in this context, a CD8^+^ T cell memory pool specific for *S*. *aureus* in healthy donors. Based on the ELISPOT results we estimate that the frequency of CD8^+^ T cells activated by *spa*-encoding mRNA accounts for a maximum of 0.04% of total CD8^+^ T cells (S6 Fig). Interestingly, this amount is equivalent to that described to be specific for influenza [47], a finding well compatible with the high exposure rates of humans to *S*. *aureus*.

Previously, Th2 responses to *S*. *aureus* were described in patients with atopic dermatitis, chronic rhinosinusitis, Hyper-IgE syndrome and asthma [48–52,44] where they were associated with allergic inflammation. Recently, it was proposed that staphylococcal serine protease–like proteins trigger a Th2 response and, thereby, prepare the grounds for allergic responses while classical virulence factors such as the alpha hemolysin are more potent in inducing Th1 responses [44]. Here, we demonstrate that two different types of staphylococcal antigens, i.e. SpA and PBP2a, trigger release of Th2-associated cytokines in CD4^+^ T cells (Fig 3 and S3 Fig), a T cell response compatible with balancing of pro- and anti-inflammatory T cell responses on skin and mucosal surfaces by bacterial commensals. Interestingly, these proteins also induced TNF and IFNγ secretion from CD4^+^ and CD8^+^ T lymphocytes (Fig 3 and S3 Fig). This is most prominently observed with SpA, while results with PBP2a were less clear because we cannot exclude effects of contaminating LPS in the PBP2a preparation (see Materials and Methods section). Strikingly, presentation of the SpA protein-derived antigen simultaneously triggered two distinct T cell pools (Th1 and Th2) as visualized by FluoroSpot analysis (Fig 5). We, thus, provide proof for the parallel existence of a Th1 and a Th2 subset of T cells responsive to the SpA antigen. This dual response could reflect ongoing control of colonization and previous response to infection.

Our data further show that T cell activation and polarization depend on the mode of antigen delivery (Fig 3). In this study, ivT mRNA delivery to MoDC was chosen to particularly activate CD8^+^ T cells because it preferentially supports loading of antigens on MHCI [21]. However, some studies suggest that later on, the translated protein is secreted and taken up by other MoDC to be presented as exogenous antigen via MHCII [25], which is the case when MoDC are pulsed with proteins. While loading of protein is more potent in inducing CD4^+^ T cell responses, CD8^+^ T cell activation seen under those conditions is likely to be due to cross-presentation. Indeed, the results indicate that the ivT mRNA approach and the adjuvant properties of the mRNA facilitate the induction of IFNγ-secreting CD8^+^ T cells by altering MoDC-derived cytokine secretion profiles (Fig 2B). This finding is well in line with the previously described innate immune recognition of RNA via cytosolic pattern recognition receptors such as retinoic acid inducible gene (RIG)-I or endosomal RNA-sensing Toll-like receptors (TLR) 7/8 [33,32] and subsequent release of TNF and type I interferon (Fig 2B). As described in other contexts, the latter is an important positive regulator of CD8^+^ T cell responses [53,54] and CD4^+^ T cell responses [55]. Here, IFN-I derived from MoDC drives the CD8^+^ T cell response to staphylococcal antigens (Fig 2D). However, our present data provide no proof of cytotoxic T lymphocyte (CTL) activity. Furthermore, MoDC are artificial and not necessarily representative of their *in vivo* counterparts in human skin and mucosa. Future work will, thus, have to elucidate the role of IFN-I in antigen presentation in the *in vivo* condition. It can only be speculated that professional interferon-producing cells such as plasmacytoid dendritic cells may play an important role in enhancing IFNγ+ T cell responses to *S*. *aureus* in the peripheral tissues [56–58]. Also, intracellular detection of *vita*PAMPs in viable bacterial cells can trigger IFN-I release from myeloid cells and support Th1 and CD8^+^ T cell responses [59].

Next, we reasoned that mRNA-mediated antigen delivery could re-polarize established Th2 responses. This is an attractive hypothesis in views of novel strategies for vaccination against pathogens whose natural immunity consists in a Th2 response. However, FluoroSpot analysis showed that the presence of mRNA increased the number of IFNγ-responsive T cells without affecting the number of Th2 cells, e.g. no reduction in IL-13-secreting cells was observed upon addition of mRNA to SpA protein and no T cell subset could be detected, which simultaneously secreted IFNγ and IL-13 upon mRNA and protein co-stimulation (Fig 5). This allows the conclusion that mRNA-mediated antigen delivery enhances the number of Th1 and CD8^+^ T cells–possibly through activation of both antigen-specific T cells and bystander T cells. However, *in vitro*, mRNA did not have the potential to alter the polarization of preexisting T cell memory subpopulations.

Next, we asked whether the T cell reactivity observed upon stimulation of T cells with antigen presenting MoDC actually resulted from preexistent antigen-specific T cell memory. Early IFNγ release from CD8^+^ T cells in overnight ELISPOT cultures, a time frame, which does not allow priming of naïve T cells but is sufficient to trigger IFNγ release from T effector and CD4^+^ central memory cells [40–42], and the increase in enriched CD45RO^+^ memory T cells supported this hypothesis (S1 Fig). Sorting of CD4^+^ and CD8^+^ T cell fractions further confirmed the presence of antigen-specific memory (Fig 4). The results showed that cytokine responses of both T cell subsets stimulated with MoDC loaded with staphylococcal protein antigens could mainly be attributed to memory T cells and priming of naïve T cells was negligible. However, with mRNA-encoded antigen naïve T cell priming induced cytokine levels equivalent to those derived of the memory cell fraction (S5 Fig), most likely accounting for the increase in IFNγ secretion observed with this mode of antigen delivery. At this point, it can only be speculated that *in vivo* the simultaneous presence of bacterial antigen and RNA might similarly potentiate the naïve and memory T cell responses to the invading pathogen.

This study further describes that presentation of *S*. *aureus* antigens to T cells triggers three cytokines exclusively seen with protein antigens e.g. G-CSF, IL-2 and IL-10 (Fig 6A). TLR2-dependent induction of IL-10 was previously found to dampen T cell responses to staphylococcal superantigen [36]. IL-2, a cytokine that promotes survival and proliferation of both T effector and T regulatory cells [60] was found to be crucial for protection against *S*. *aureus* infection after vaccination in a retrospective analysis of clinical study materials [39]. Additionally, secretion and beneficial effects of G-CSF have previously been described in the resolution of *S*. *aureus* infection [61–64]. To better understand the role of these cytokines we performed MoDC/T cell co-culture experiments in the presence and absence of these cytokines. Interestingly, G-CSF—but not IL-10 nor IL-2—prevented early IFNγ release in response to mRNA-encoded antigens (Fig 6B and 6C). This result emphasizes a potential role of G-CSF in the regulation of anti-staphylococcal T cell memory. It is supported by earlier reports that claimed that G-CSF favors Th2 cell responses and/or induction of T regulatory and anergic T cells. These studies reported direct effects on T cells and indirect modulation of antigen presenting cell function [37,38,65,66]. G-CSF could, thus, play a central role in preventing inflammatory responses to colonizing *S*. *aureus*.

Despite the attractiveness of this hypothesis this observation highlights the limitations of an *in vitro* study. Although the technical approach allows a reliable assessment of frequency and quality of staphylococcal antigen-specific T cell responses at a given time point, this snapshot and many findings in regards to T cell subsets and cytokines remain speculative in views of their actual role and significance *in vivo*. In some circumstances *in vitro* analysis can predict in *vivo* responses [69]. However, in all cases *in vivo* proof-of-concept is required for exploiting these findings for therapeutic purposes. Future work will be dedicated to define the *in vivo* correlates of protection in *S*. *aureus* infection and colonization. In the absence of functional evidence *in vivo* no definite statement can be made on the role of G-CSF and Th1 or Th2/Treg profiles in promotion of tolerance on colonized skin and mucosa and/or protection or aggravation of infection.

The Th2/Treg-biased T cell repertoire against two tested *S*. *aureus* protein antigens observed in this study could be beneficial for commensalism. This type of immune response favors the immune evasion typically seen with this pathogen. However, it might hamper the generation of potentially protective Th1 and CD8^+^ T cell responses. In views of the accumulation of resistance genes in MRSA strains alternatives to antibiotics need to be considered. Interestingly, we observed that Th2 and Treg-associated cytokines were induced by the same antigens. This stands in contrast to a recent study where human Th2 cells and Tregs were responsive to different allergens [67]. At this point we cannot differentiate whether different peptides derived from the same antigen are specifically recognized by distinct T cell subsets.

Albeit SpA has been suggested as a vaccine antigen [68], to our knowledge, human T cell responses towards SpA have not yet been described. Notably, the response was superior to that elicited by the MRSA-specific PBP2a or the lipoprotein SitC, which confirms a previous report [43]. This might be due to the fact that SpA is expressed in high quantity on the cell wall of 99% of *S*. *aureus* strains [70,71], while contact of SitC and PBP2a to immune cells is restricted due to their subcellular localization within the cytoplasmic membrane or the peptidoglycan layer, respectively [72,73]. On the one hand the high levels of surface expression of protein A might explain its immunogenicity in regards to formation of T cell memory responses. On the other hand we cannot fully exclude that immune modulatory properties of SpA on MoDC such as binding of residual immunoglobulin bound by Fc receptors or activation of TNFR1 [74] could modulate the MoDC or T cell response. However, release of Th2 and Treg associated cytokines is not characteristic for TNFR1 engagement, which normally leads to cell death and inflammation [75]. Thus, our findings confirm earlier reports that protein A (SpA and *spa* mRNA) might be suitable as a vaccine antigen [68] but the simultaneous induction of Th1, Th2 and Treg responses adds additional complexity to vaccine design. In particular, unwanted enhancement of Th2/Treg responses by vaccination might not be suitable to prevent infection.

Considering that mRNA-based vaccines are successfully applied in malignancy, e.g. in an immunosuppressory condition where tumor antigens are recognized as *self*, they might also be efficient in fighting commensals. In support of use of IFN-I inducing adjuvants such as TLR7 and RIG-I ligands in vaccines against *S*. *aureus* [8], our data highlight the crucial role of IFNα and TNF in eliciting inflammatory T cell responses, e.g. production of TNF and IFNγ (Fig 2). However, the activation of bystander T cells that are not specific for the antigen by mRNA cannot be excluded (Fig 1), nor are there tools that allow us to quantify the number of T cells that are antigen-specific, which is mainly due to the unknown T cell epitopes required for tetramer staining. Thus, the ability of mRNA to prime naïve T cells (S5 Fig) needs to be interpreted with care. However, taking into account that mRNA activates naïve T cells, mRNA—next to acting as a Th1-polarizing adjuvant—could represent an important new component in the development of protective vaccine formulations against *S*. *aureus*. Nonetheless, our experiments were exclusively carried out *in vitro* and future work is needed to evaluate the potential of mRNA-based antigen delivery *in vivo*.

## Materials and methods

### Ethics statement

The use of human peripheral blood mononuclear cells (PBMC) from buffy coats was approved by the local institutional review board (Ethics committee of the Medical Faculty of the University of Frankfurt, Germany, #154/15). Buffy coats of anonymized healthy donors were obtained from the German Red Cross South transfusion center (Frankfurt am Main, Germany).

### Reagents

Protein stimulation of cell culture was carried out at 1 μg/ml using protein A (SpA, isolated from *S*. *aureus* Cowan Strain I (SAC, GE Healthcare, Uppsala, Sweden), recombinant PBP2a (Antikörper-online GmbH, Aachen, Germany), SitC isolated from *S*. *aureus* as described below, tetanus toxoid (TT, Statens serum institute, Copenhagen, Denmark) or 1 μl/well Influenza H1N1 MP1 peptide pool (Miltenyi Biotech, Bergisch-Gladbach, Germany). This peptide pool consists of 15-mer sequences covering the whole MP1 sequence and bears the potential to trigger both CD4^+^ and CD8^+^ T cells responses.

All microbeads in this study used for cell isolation were obtained from Miltenyi Biotech (Bergisch-Gladbach, Germany).

Blocking antibodies anti-IFNα (#21100–1), anti-TNFα (#MAB210), isotype control anti-mouse IgG_1_ (#MAB002) were purchased from R&D Systems, Inc. (Minneapolis, U.S.).

Recombinant cytokines (IL-4, granulocyte macrophage colony-stimulating factor (GM-CSF), G-CSF, IL-2, IL-10) were purchased from Miltenyi Biotech, (Bergisch-Gladbach, Germany).

Antibodies used for sorting and/or purity determination by flow cytometry or were all from BD Biosciences, Heidelberg, Germany: anti-CD14-V450, anti-CD83-APC, anti-CD11c-PE and anti-HLA-DR-PerCP-Cy5.5 (for phenotyping MoDC), anti-CD4-PerCP-Cy-5.5, anti-CD8-PE, anti-CD45RO-APC (for purity determination), anti-CD45RA-PE, anti-CD45RO-APC-H7, anti-CD8-APC (for purification).

### Bacterial strains and DNA isolation

Genomic DNA was isolated from *S*. *aureus* SAC (DSMZ #20372, Braunschweig, Germany) and USA300 using the UltraClean Microbial DNA Isolation Kit (MoBio, Dianova, Hamburg, Germany).

### *in vitro* transcription

Full-length *spa* (from SAC), *sitC* and *mecA* (from USA300) were cloned into pT7CFE1-cMyc (Thermo Fisher, Dreieich, Germany) using KpnI and XhoI cutting sites. The control firefly *luciferase* gene was amplified from the plasmid pGL3-basic (Promega GmbH, Mannheim, Germany) and cloned into pT7CFE1-cMyc at XhoI and NotI restriction sites. *In vitro* transcription (ivT) was performed with mMESSAGE mMachine T7 Kit (Ambion,Thermo Fisher, Paisley, UK) following the manufacturer’s protocol. In brief, the original pT7CFE1-cMyc plasmid (for non-coding (NC) mRNA control) or the plasmid carrying *spa*, *mecA*, *sitC* or *luc* gene was linearized. The transcription reaction mix containing the cap analogue m^7^G(5')ppp(5')G was incubated with the linearized plasmid for 2 h at 37°C. Following DNAse treatment, mRNA was recovered using lithium chloride precipitation. mRNA quality was controlled using the Experion RNA StdSens analysis Kit (Bio-Rad Laboratories GmbH, Munich, Germany) and quantified with a Quantus Fluorometer (Promega GmbH, Mannheim, Germany).

To confirm mRNA translation and functional integrity of the resultant proteins after ivT mRNA transfection HEK293 cells and MoDC were transfected with *luciferase* mRNA and luciferase activity was determined as described below. Next, mRNA encoding *spa* was transfected into HEK293 cells and intracellular translation of SpA was visualized via binding of IgG to SpA protein (see below).

### Luciferase assay

To confirm mRNA translation and protein integrity after ivT, HEK293 cells and MoDC were transfected with *luciferase* mRNA. Human embryonic kidney 293 cells (HEK293) were obtained from DSMZ (#ACC-305, Braunschweig, Germany). Cleavage of luciferin by the luciferase confirmed successful translation of *luc* mRNA in HEK293 cells. Light emission increased with increasing amounts of transfected *luc* mRNA in HEK293 cells (S7A Fig, left panel) and MoDC (S7A Fig, right panel).

HEK293 cells were regularly tested for *Mycoplasma* contamination by PCR.

HEK293 cells or MoDC were seeded at a density of 5*10^5^ cells/ml in RPMI 1640 (Gibco, Life science, Darmstadt, Germany) supplemented with 10% FCS (Sigma-Aldrich Chemie GmbH, Munich, Germany), 1% penicillin/streptomycin (10.000 IU/ml and 10.000 μg/ml and, 1% 200 mM L-Glutamine (all from Biochrom AG, Berlin, Germany) in flat bottom 96 well plates (Greiner Bio-One GmbH, Frickenhausen, Germany). Cells were transfected with the indicated amounts of ivT *luc* mRNA complexed with lipofectamine 2000 (Invitrogen, Karlsruhe, Germany). 0.25 μl Lipofectamine 2000 were complexed with 100 ng mRNA in 50 μl OptiMEM. One hour post transfection, 150 μl culture medium were added per well. Luciferase activity was quantified 24 h (HEK293 cells) or 4 h (MoDC) post transfection using the Dual-Luciferase Reporter Assay System kit (Promega GmbH, Mannheim, Germany) according to the protocol provided by the manufacturer with the following adaptions: cells were lysed by adding 40 μl PBL buffer for 15 min shaking at RT; 40 μl of cell lysate was mixed with 40 μl of LARII substrate and light emission was measured immediately with a Wallac Victor^2^ 1420 multilable counter (PerkinElmer, Waltham, U.S.)

### Western blot

HEK293 cells were plated at a density of 5*10^5^ cells/ml in RPMI 1640 (Gibco by Life science, Darmstadt, Germany), supplemented with 10% FCS (Sigma-Aldrich Chemie GmbH, Munich, Germany), 1% penicillin/streptomycin (10.000 IU/ml and 10.000 μg/ml and, 1% 200 mM L-glutamine (all from Biochrom AG, Berlin, Germany) in 96 well plates (Greiner Bio-One GmbH, Frickenhausen, Germany). Cells were transfected with 100 ng *spa* or 100 ng NC mRNA complexed with 0.25 μl lipofectamine 2000 (Invitrogen, Karlsruhe, Germany). One hour post transfection fresh medium was added and cells incubated for additional 24 h. Thereafter cells were lysed by adding 5 x SDS lysis buffer, quadruplicates pooled and lysates cooked at 96°C for 10 min. Proteins were separated by SDS-PAGE. After semidry blotting the nitrocellulose membrane was blocked with TBS/5% dry milk and incubated with IVIg 1:2000 (Octapharma GmbH, Langenfeld, Germany) and HRP-conjugated goat anti-human Ig (Jackson ImmunoResearch, UK) to visualize the binding of IgG to the Ig-binding domains of the SpA protein immobilized on the membrane (S7B Fig).

### Purification of SitC

SitC (MntC) is a lipoprotein in USA300 and has been widely used as a model protein for immune stimulation. Originally it was thought to be involved in iron transport but later it was found to bind manganese ions and was renamed as MntC [73]. To avoid confusion we keep here the original designation, SitC.

The purification of SitC from the membrane of *S*. *aureus* SA113 carrying pTX30SitC-his was carried out following the previous study [27] with a small modification. Briefly, the clone was pre-cultivated aerobically at 37°C in 3 l of the B-medium without glucose until OD_578nm_ 0.5, then supplied with 0.5% xylose for 15 h for induction of SitC expression. The bacterial cells were harvested by centrifugation at 4000 x g at 4°C for 20 min and washed two times with Tris buffer (20 mM Tris, 100 mM NaCl, pH 8.0). Then the pellet was resuspended with 100 ml of Tris buffer containing protease inhibitor table (Merck, Darmstadt, Germany), DNAse (10 μg/ml) and lysostaphin (30 μg/ml) and incubated at 37°C for 3 h to disrupt the cell wall. After the first ultracentrifugation (235,000 x g for 45 min at 4°C), membrane proteins were dissolved overnight at 6°C with Tris buffer containing 2% Triton X100. After another ultracentrifugation step, the supernatant containing the membrane fraction was incubated with 1ml of Ni-NTA super flow beads (Qiagen, Germany) overnight at 6°C under mild rotation (20 rpm). One volume of Ni-NTA beads were washed four times with 20 volumes of washing buffer (Tris buffer containing 0.25% TritonX-100 and 20 mM imidazole), subsequently the beads were washed two times with 20 volumes of the same buffer containing 40 mM imidazole and finally SitC was eluted with 10 ml of the same buffer containing 500 mM imidazole. SitC was concentrated via centrifugal ultra-filter unit with a molecular mass cut-off of 10 kDa (Sartorius AG, Göttingen, Germany). Finally, the SitC purification was checked by SDS-PAGE and the total protein amount was determined by a Bradford assay kit.

### Endotoxin measurements

Purified proteins were tested for endotoxin contamination using the Endosafe-PTS System (Charles River, U.S.). Endotoxin levels for 1 μg/ml protein solutions were <0.005 EU/ml for SpA, 0.006 EU/ml for SitC and 0.121 EU/ml for PBP2a.

### Generation of monocyte-derived dendritic cells

PBMC were isolated by Pancoll gradient centrifugation (PAN-Biotech, Aidenbach, Germany). Monocytes were isolated by positive selection with anti-CD14 microbeads. The purity was analyzed by flow cytometry on a FACS LSRII SORP (BD Biosciences, Heidelberg, Germany) with anti-human CD14 and ranged from 90%-99% (S8B Fig). Remaining PBMC were frozen in RPMI 1640 supplemented with 20% FCS and 20% DMSO (both from Sigma-Aldrich, Munich, Germany) for subsequent isolation of autologous T cells.

Isolated monocytes were counted using trypan blue exclusion (Applichem Panreac, Darmstadt, Germany) and seeded at a density of 1.5*10^6^ cells/ml in RPMI 1640 (Gibco, Life science, Darmstadt, Germany), supplemented with 10% FCS (Sigma-Aldrich Chemie GmbH, Munich, Germany), 1% penicillin/streptomycin, 1% L-Glutamine and 1% HEPES buffer (all from Biochrom, Berlin, Germany), 50 μM 2-Mercaptoethanol (Sigma-Aldrich, Munich, Germany), 50 ng/ml human GM-CSF and 20 ng/ml human lL-4. Cells were incubated at 37°C and 5% CO_2_, medium exchanged on after 3 days and cells harvested on day 6. Successful differentiation of monocytes into monocyte-derived dendritic cells (MoDC) (CD14^-^, HLA-DR^high^, CD11c^+^, CD83^dim^) was confirmed by flow cytometric analysis (S8A Fig).

### Isolation of autologous T cells

For isolation of autologous T cells frozen PBMC were thawed on day 6 of MoDC culture and T cells isolated via positive selection with anti-CD4, anti-CD8 or anti-CD45RO microbeads. The purity was analyzed by flow cytometry with anti-human CD4, anti-CD8 or anti-CD45RO and ranged from 85%-98% (S8C and S8B Fig).

### Sorting of T cell subsets by flow cytometry

Following isolation of CD4^+^ or CD8^+^ cells, respectively via magnetic beads from freshly thawed PBMC and recovery of the T cells overnight in 10% human serum (Biochrom AG, Berlin, Germany), cells were labeled with anti-CD45RA, anti-CD45RO, anti-CD14 and anti-CD8 antibodies and purified trough 85 μm nozzle by BD FACSAria Fusion (BD Biosciences, Heidelberg, Germany) using the BD FACS Diva software version 8.0.1. T cell subsets were sorted as either CD8^-^ or CD8^+^ and, CD45RA^+^CD45RO^-^CD14^-^ (naïve T cells) and CD45RA^-^CD45RO^+^CD14^-^ (memory T cells) by flow cytometric analysis (S9 Fig). Purity of sorted T cells was confirmed by re-analysis and ranged from 85–99% (S8E and S8F Fig).

### Stimulation and transfection of MoDC and co-culture experiments with PBMC or T cells

MoDC were harvested by washing cells vigorously with DPBS (Gibco, Life science, Darmstadt, Germany). 2*10^4^ MoDC were plated in 50 μl/well RPMI 1640 (Gibco by Life science, Darmstadt, Germany), supplemented with 1% penicillin/streptomycin (10.000 IU/ml and 10.000 μg/ml), 1% 200 mM L-Glutamine (all from Biochrom AG, Berlin, Germany) and 5% Xerumfree (TNC Bio, Eindhoven, Netherlands).

Transfection with ivT mRNA was performed using in 50 μl Opti-MEM medium/ well (Gibco by Life science, Darmstadt, Germany) containing 100 ng mRNA complexed with 0.25 μl lipofectamine 2000 (Invitrogen, Karlsruhe, Germany). Where indicated, 1 ng/ml human recombinant IL-2, IL-10 or G-CSF or 2,5 μg/ml blocking antibodies anti-IFNα or anti-TNFα were added to MoDC 1 h after transfection. Alternatively, MoDC were stimulated with SpA, PBP2a, SitC, tetanus toxoid or 1 μl/well Influenza H1N1 MP1 peptide pool. After 1 hour 1*10^5^ autologous PBMC, CD4^+^ or CD8^+^ T cells were added to MoDC (final volume 200 μl/ well). Co-cultures were incubated for 16 h at 37°C and 5% CO_2_.

### Cytokine measurements

#### ELISPOT and FluoroSpot

For ELISPOT assays, MoDC/T cell or MoDC/PBMC co-cultures were performed in 96-well MultiScreen HTS IP plates (0.45 μm, clear, Merck Chemicals GmbH, Darmstadt, Germany), coated with anti-IFNγ capture antibody (BD Biosciences, Heidelberg, Germany) overnight at 4°C. Following blocking for two hours at room temperature with culture medium, cells were seeded and stimulated as described above. After 16 h incubation, cells were discarded and plates washed in sterile water and PBS / 0.05% Tween20 (Sigma-Aldrich Chemie GmbH, Munich, Germany). Biotinylated anti-human IFNγ detection antibody (BD Biosciences, Heidelberg, Germany) was added in PBS / 10% FCS and plates incubated for two hours. After washing in PBS / 0.05% Tween20 Alkaline Phosphatase (AP)-conjugated Streptavidin (BD Biosciences, Heidelberg, Germany) was added 1:1000 in PBS/10% FCS followed by incubation for one hour. Development of the plate was performed with the AP conjugate substrate kit (Bio-Rad Laboratories GmbH, München, Germany); the reaction was stopped with water and the plate dried overnight.

FluoroSpot assay was carried out using the MabTech Kit (IFNγ/IL-13, MabTech, Stockholm, Sweden) following the provided protocol.

Spots and enzymatic activity were quantified with an iSpot FluoroSpot Reader System (AID, Strassberg, Germany).

#### ELISA and Luminex technology

Cytokine secretion was quantified in supernatants from MoDC alone or 5 day-MoDC/T cell co-cultures. The Milliplex Human T_h_17 Magnetic Bead Panel Kit (Merck Millipore, Darmstadt, Germany) was used to analyze cytokine production in the supernatants of 0.5 x 10*6/ml MoDC stimulated with mRNA or proteins for one or four days, respectively; T cell cytokines were quantified using the Bio-Plex Pro Human Cytokine 17-plex Assay (Bio-Rad Laboratories GmbH, Munich, Germany). Measurements for both were carried out on MagPix XMAP technology (Luminex, Austin, U.S.). Of note, to minimize donor-dependent variability in the latter experiments we only included donors where overnight IFNγ responses in ELISPOT exceeded those obtained with the NC mRNA control.

IFNα was quantified by ELISA (human IFNα Module Set, eBioscience, San Diego, U.S.).

### Statistical analysis

Statistical analysis of results was carried out using GraphPad Prism 7.01 (Graphpad Software Inc. San Diego, USA). Following testing for normal distribution, paired two-tailed Student’s t-test was used treating samples as paired data points. If normal distribution was not applicable, non-parametric Wilcoxon matched-pairs signed rank test was used to calculate significance of two groups, one-way ANOVA for testing multiple groups. Each experiment was carried out at least in duplicates. Results are presented as mean values ± standard error of the mean and were considered statistically significant at * p< 0.05, ** p< 0.01, *** p< 0.001 and **** p< 0.0001. p≥ 0.05 = not significant (n.s.).

## Supporting information

S1 Fig T cell activation by delivery of ivT antigens. **(a)** IFNγ production in CD4^+^ (upper panel) and CD8^+^ (lower panel) T cell cultures upon exposure to staphylococcal antigens delivered via mRNA, e.g. non-coding mRNA (NC), *spa*, *mecA* and *sitC* or with lipofectamine (LF) alone. IFNγ production of one representative donor is shown in duplicates. **(b)** Comparison of IFNγ production in CD8^+^ and CD45RO^+^ T cells stimulated with NC or *spa* mRNA-transfected MoDC. ELISPOT wells of one representative donor out of at least 8 experiments are depicted in duplicates.
(TIF)

S2 Fig Cytokine production of MoDC stimulated with ivT mRNA or protein of staphylococcal antigens. Levels of IL-1β and IL-10 are displayed as mean values ± SEM of n = 4 donors detected by multiplex cytokine assay after one day of culture. n.d. not detected. Lipofectamine (LF) alone, non-coding mRNA (NC) and a peptide pool from matrix protein 1 (MP1) of H1N1 Influenza virus and Tetanus toxoid (TT) served as controls. Experiments were carried out using technical duplicates.
(TIF)

S3 Fig T cell cytokine profiles in response to staphylococcal mRNA and protein antigens. Cytokine secretion profiles in day 5 supernatants of CD4^+^ (left panel) or CD8^+^ T cells (right panel) stimulated with mRNA or protein antigens was performed using a multiplex cytokine array: (a) Th1/Th17 cytokines: IL-17a, IFNγ, TNF and (b) Th2 cytokines: IL-4, IL-5, IL-13. The graphs depict the mean values ± SEM obtained by n = 6 independent donors in technical duplicates.
(TIF)

S4 Fig Cytokine production of MoDC/T cell co-cultures stimulated with *S. aureus* proteins and ivT mRNA. Multiplex assay was performed with 5 days supernatants of mRNA or protein-stimulated CD4^+^ (upper panel) or CD8^+^ T cells (lower panel) and IL-6 and IL-8 were measured. n = 6 different donors (analyzed in technical duplicates) were analyzed and displayed as mean values ± SEM.
(TIF)

S5 Fig mRNA-derived antigens activate naïve and memory T cells. Human CD4^+^ **(a)** or CD8^+^ **(b)** T cells were isolated from frozen PBMC via magnetic beads. Cell fractions were either purified for CD14^-^CD8^-^ **(a)** and CD14^-^CD8^+^ **(b**) and CD45RO^-^CD45RA^+^ (naïve) and CD45RO^+^CD45RA^-^ (memory) phenotype. Cytokine secretion profiles after 5 days of MoDC/T cell co-culture loaded with *spa* mRNA were measured by multiplex cytokine array. TNF, IFNγ, IL-5 and IL-13 are presented as mean ± SEM of at least n = 7 donors. Student’s paired t-test was used to determine significance. p value refers to the same condition in the unstimulated control. p**<0.01, p*< 0.05, n.s. not significant.
(TIF)

S6 Fig Calculation of *S. aureus*-specific T cells. Based on the ELISPOT results (see Fig 1) the percentage of IFNγ secreting T cells was estimated by the number of spots detected by ELISPOT. For calculation of the cells triggered by mRNA stimulation, the background NC was subtracted, for the cells activated by proteins, the unstimulated control was subtracted. Results are displayed as mean ± SEM of n = 8 donors.
(TIF)

S7 Fig Translation control of *in vitro* transcribed mRNA. **(a)** HEK293 cells (left) and MoDC (right) were transfected with the indicated amounts of *luc* (luciferase) mRNA. Luminescence activity was measured with a luminescence plate reader in n = 2–3 independent experiments. The results obtained from triplicates are shown as mean values ± SEM. p****< 0.0001 (paired t-test) **(b)** Translation of ivT *spa* mRNA was analyzed by Western blot from lysates of transfected HEK293 cells. Data are representative out of two independent experiments.
(TIF)

S8 Fig Purity of cell subsets. **(a)** Following differentiation of CD14^+^ monocytes, MoDC were generated for 6 days via IL-4 and GM-CSF. After 6 days, cells were harvested and MoDC phenotype was confirmed by flow cytometry using anti-CD14-V450, anti-CD83-APC, anti-CD11c-PE and anti-HLA-DR-PerCP-Cy5.5. Histograms of monocytes, corresponding MoDC and unstained negative control are shown of one representative donor out of at least six independent experiments. Purity of **(b)** CD14^+^ monocytes **(c)** CD4^+^ T cells, **(d)** CD8^+^ T cells after AutoMACS isolation was determined by flow cytometry using anti CD14-V450, anti-CD4-PerCP-Cy-5.5 and anti-CD8-PE, respectively. Purity of purified naïve and memory **(e)** CD4^+^ and **(f)** CD8^+^ T cells was confirmed by flow cytometry with anti-CD45RO-APC-H7 and anti-CD45RA-PE. Results are shown as histograms or dot plots of one representative donor.
(TIF)

S9 Fig Gating strategy for FACS sorting. Following isolation of **(a)** CD4^+^ or **(b)** CD8^+^ cells via magnetic beads from PBMC, T cells were labeled with anti-CD45RA-PE, anti-CD45RO-APC-H7, anti-CD14-V450 and anti-CD8-APC antibodies and purified by BD FACSAria Fusion. First, doublets were excluded and cells gated for viable lymphocytes. Following exclusion CD14^+^ cells, naïve (CD45RA^+^CD45RO^-^) and memory (CD45RA^-^CD45RO^+^) T cells were sorted. Analysis was carried out with the BD FACS Diva software version 8.0.1. Data are representative out of at least 3 independent experiments.
(TIF)
