# Supplementary figures and images for: Antigen delivery to dendritic cells shapes human CD4+ and CD8+ T cell memory responses to Staphylococcus aureus
Source: PLoS Pathog. 2017 May 25;13(5):e1006387. doi: 10.1371/journal.ppat.1006387 (PMC5444865; doi:10.1371/journal.ppat.1006387)

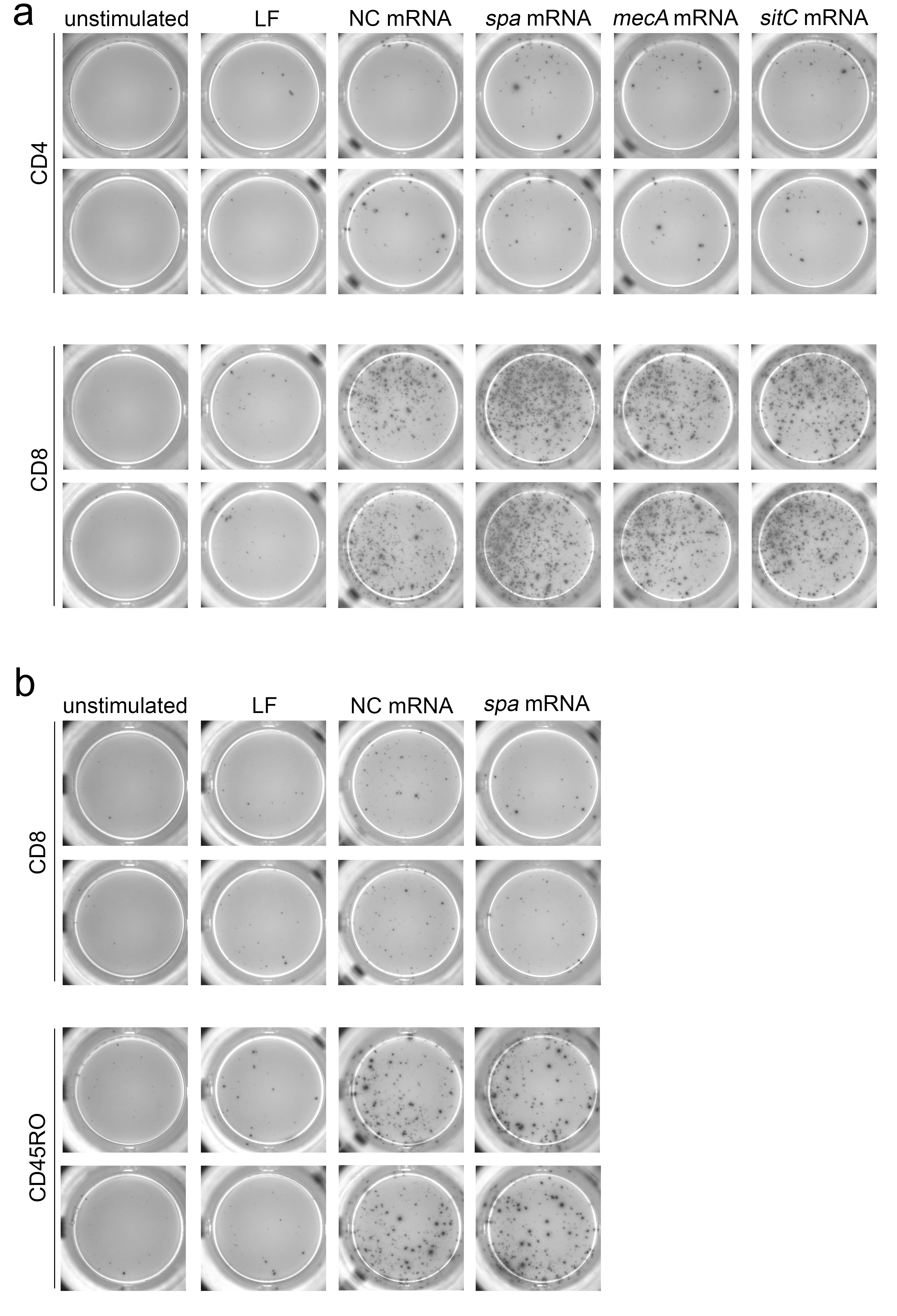

Supplement: S1 Fig — (a) IFNγ production in CD4+ (upper panel) and CD8+ (lower panel) T cell cultures upon exposure to staphylococcal antigens delivered via mRNA, e.g. non-coding mRNA (NC), spa, mecA and sitC or with lipofectamine (LF) alone. IFNγ production of one representative donor is shown in duplicates. (b) Comparison of IFNγ production in CD8+ and CD45RO+ T cells stimulated with NC or spa mRNA-transfected MoDC. ELISPOT wells of one representative donor out of at least 8 experiments are depicted in duplicates. (TIF) [file ppat.1006387.s001.tif]

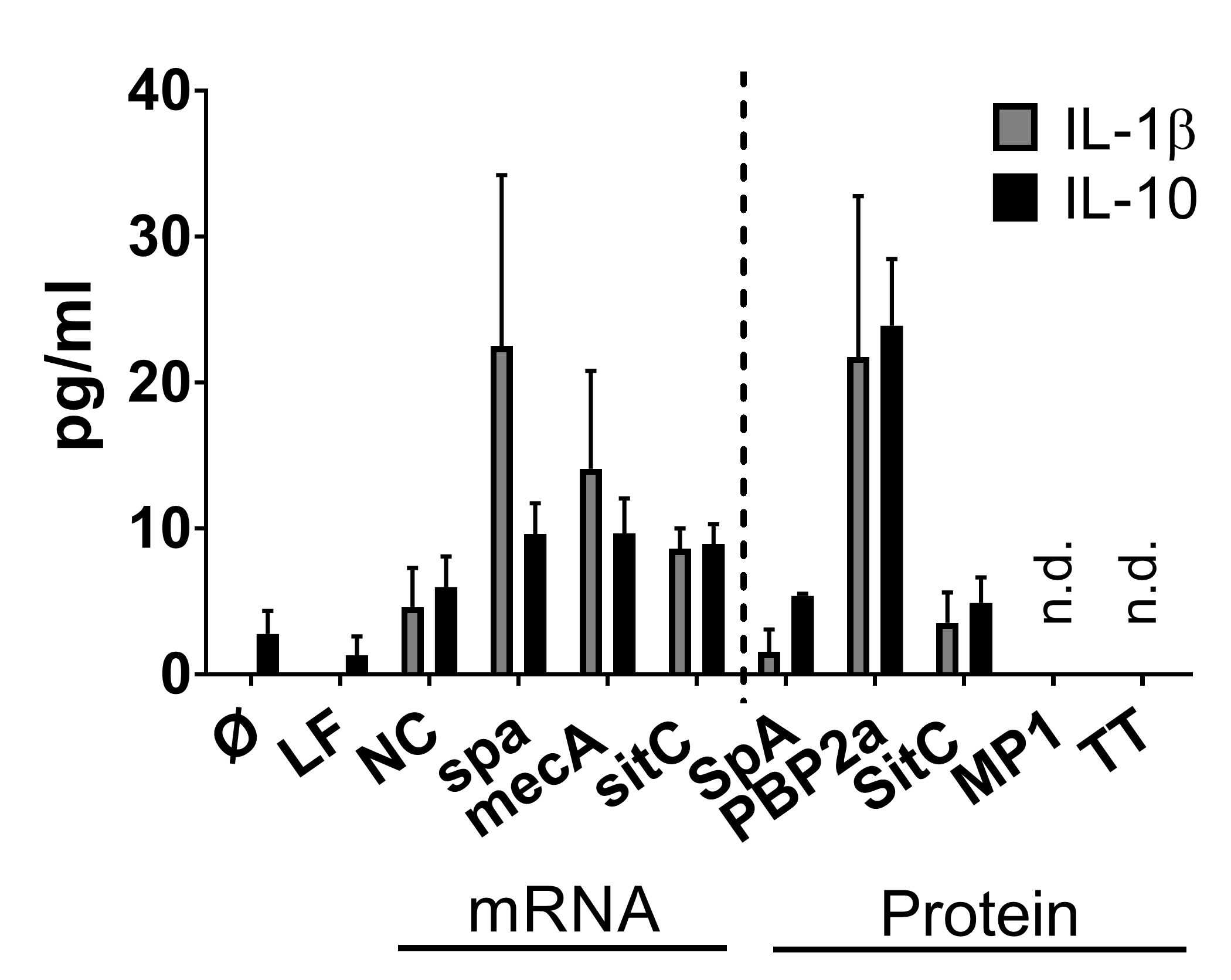

Supplement: S2 Fig — Levels of IL-1β and IL-10 are displayed as mean values ± SEM of n = 4 donors detected by multiplex cytokine assay after one day of culture. n.d. not detected. Lipofectamine (LF) alone, non-coding mRNA (NC) and a peptide pool from matrix protein 1 (MP1) of H1N1 Influenza virus and Tetanus toxoid (TT) served as controls. Experiments were carried out using technical duplicates. (TIF) [file ppat.1006387.s002.tif]

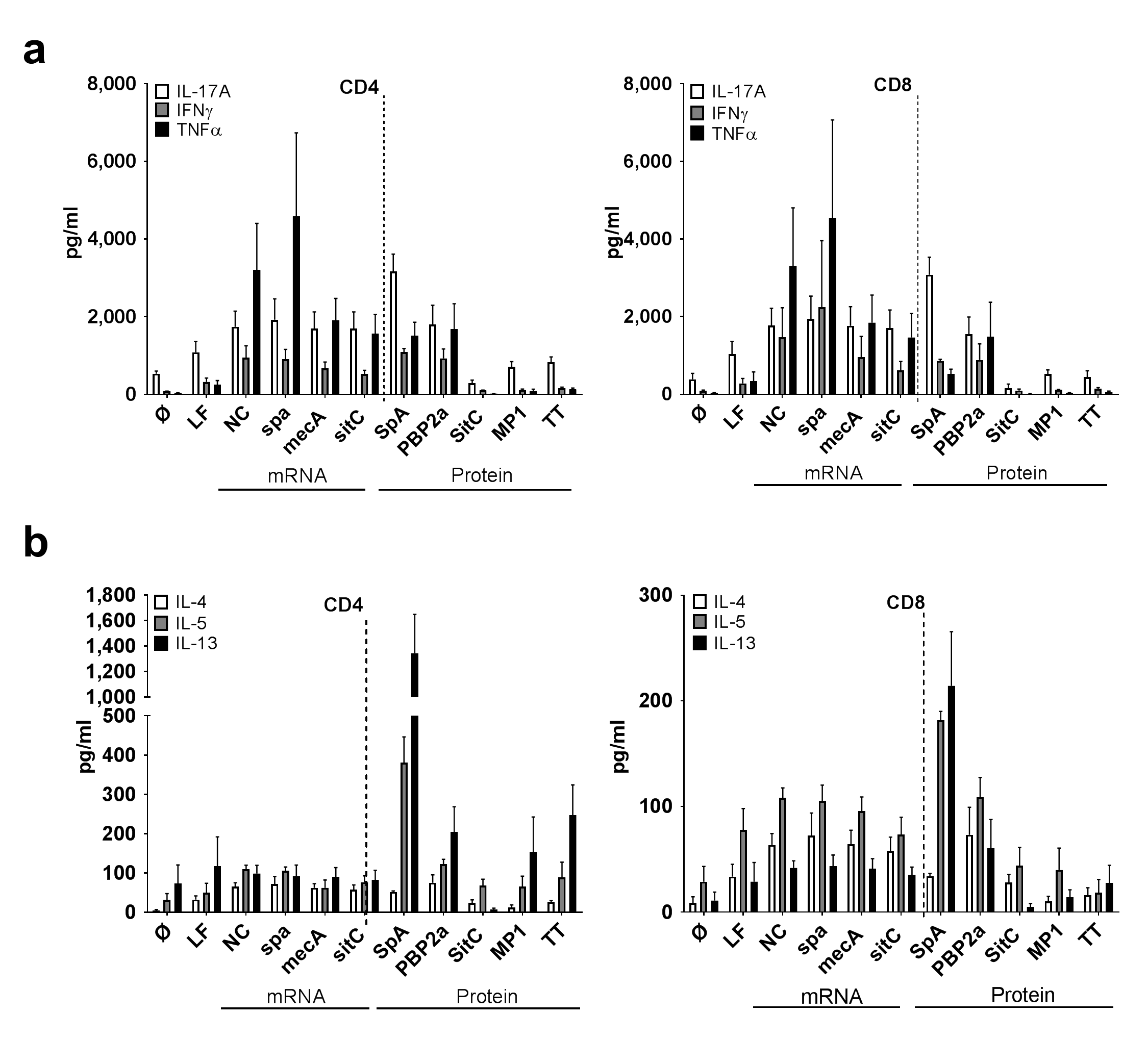

Supplement: S3 Fig — Cytokine secretion profiles in day 5 supernatants of CD4+ (left panel) or CD8+ T cells (right panel) stimulated with mRNA or protein antigens was performed using a multiplex cytokine array: (a) Th1/Th17 cytokines: IL-17a, IFNγ, TNF and (b) Th2 cytokines: IL-4, IL-5, IL-13. The graphs depict the mean values ± SEM obtained by n = 6 independent donors in technical duplicates. (TIF) [file ppat.1006387.s003.tif]

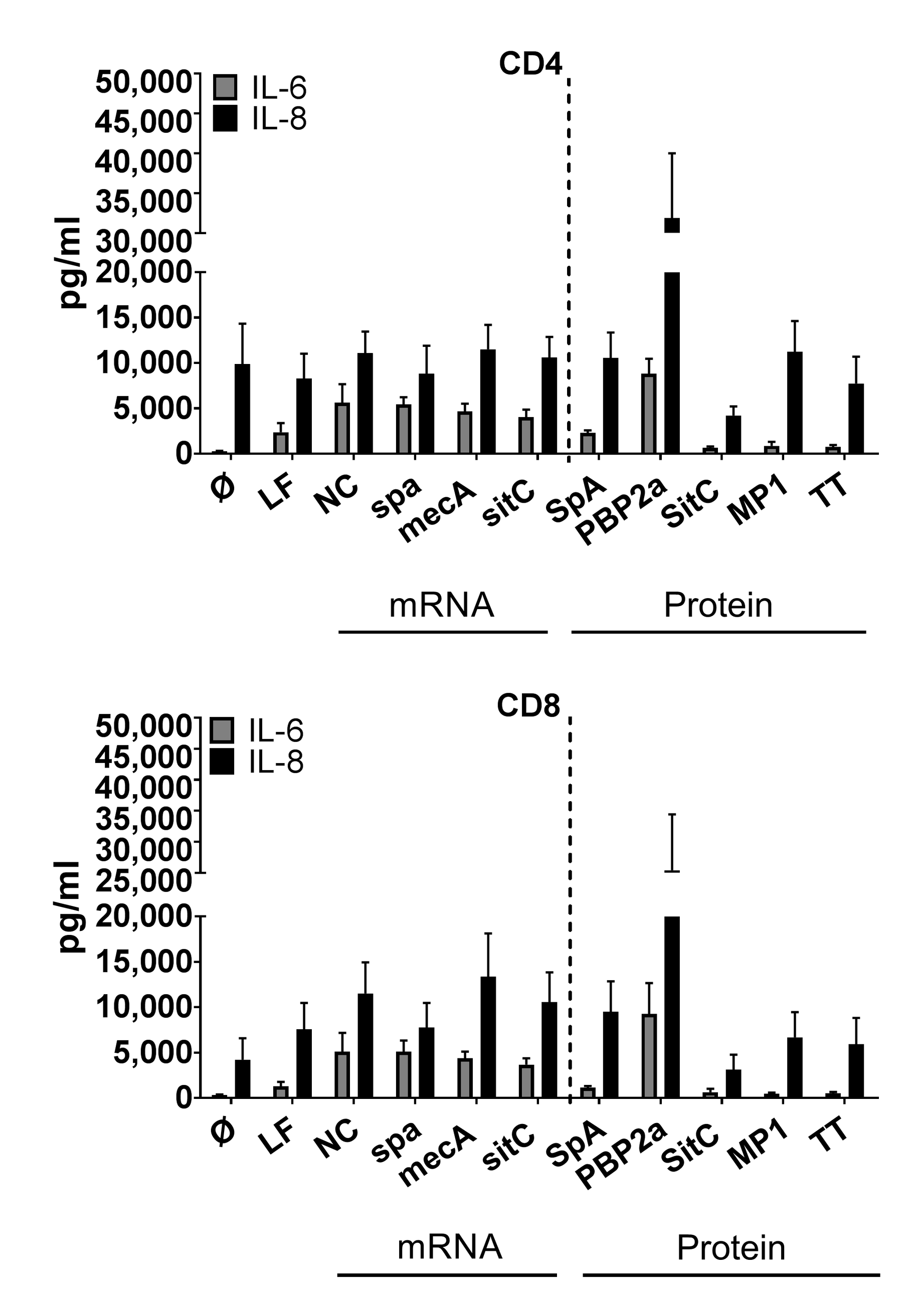

Supplement: S4 Fig — Multiplex assay was performed with 5 days supernatants of mRNA or protein-stimulated CD4+ (upper panel) or CD8+ T cells (lower panel) and IL-6 and IL-8 were measured. n = 6 different donors (analyzed in technical duplicates) were analyzed and displayed as mean values ± SEM. (TIF) [file ppat.1006387.s004.tif]

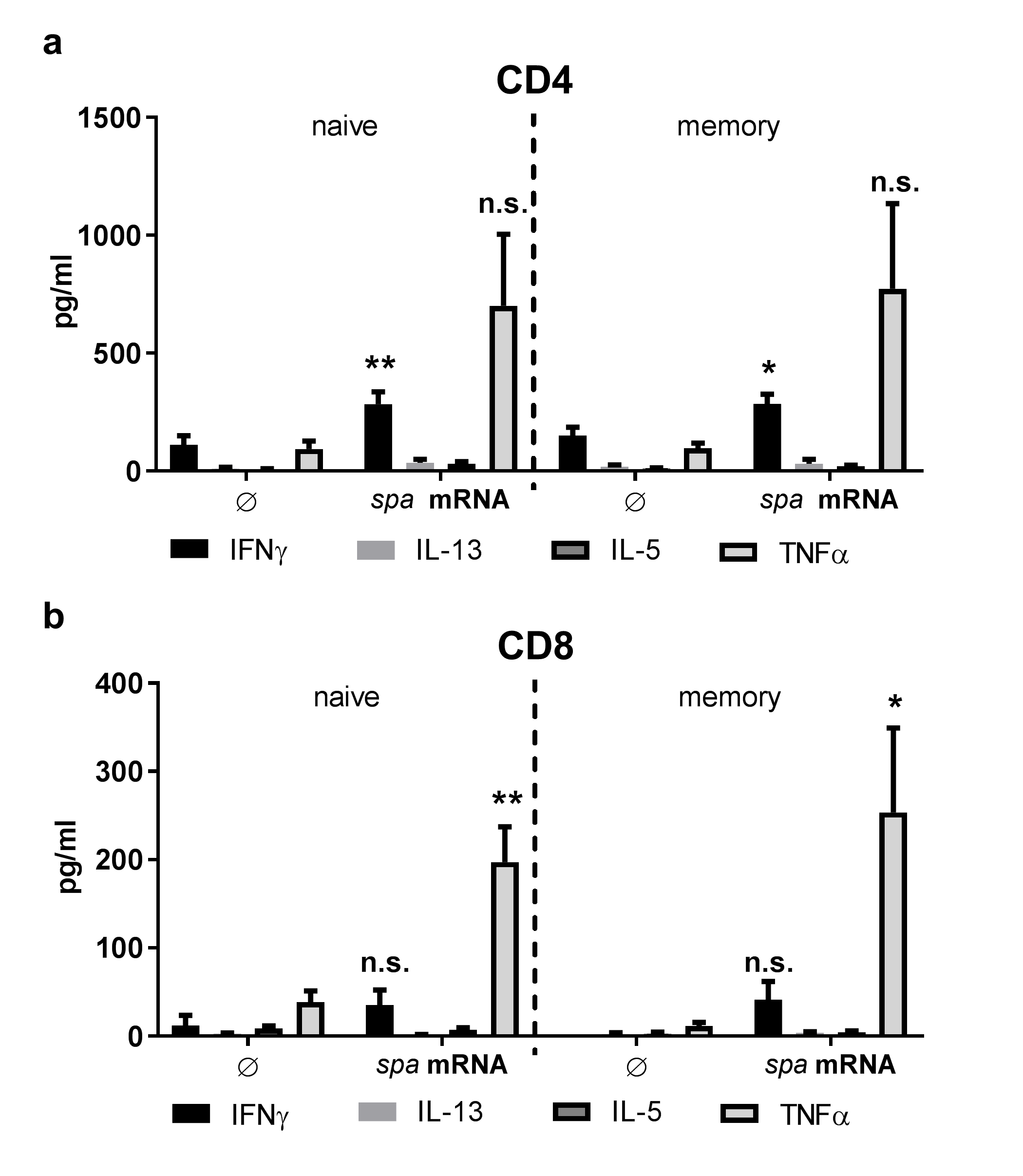

Supplement: S5 Fig — Human CD4+ (a) or CD8+ (b) T cells were isolated from frozen PBMC via magnetic beads. Cell fractions were either purified for CD14-CD8- (a) and CD14-CD8+ (b) and CD45RO-CD45RA+ (naïve) and CD45RO+CD45RA- (memory) phenotype. Cytokine secretion profiles after 5 days of MoDC/T cell co-culture loaded with spa mRNA were measured by multiplex cytokine array. TNF, IFNγ, IL-5 and IL-13 are presented as mean ± SEM of at least n = 7 donors. Student’s paired t-test was used to determine significance. p value refers to the same condition in the unstimulated control. p**<0.01, p*< 0.05, n.s. not significant. (TIF) [file ppat.1006387.s005.tif]

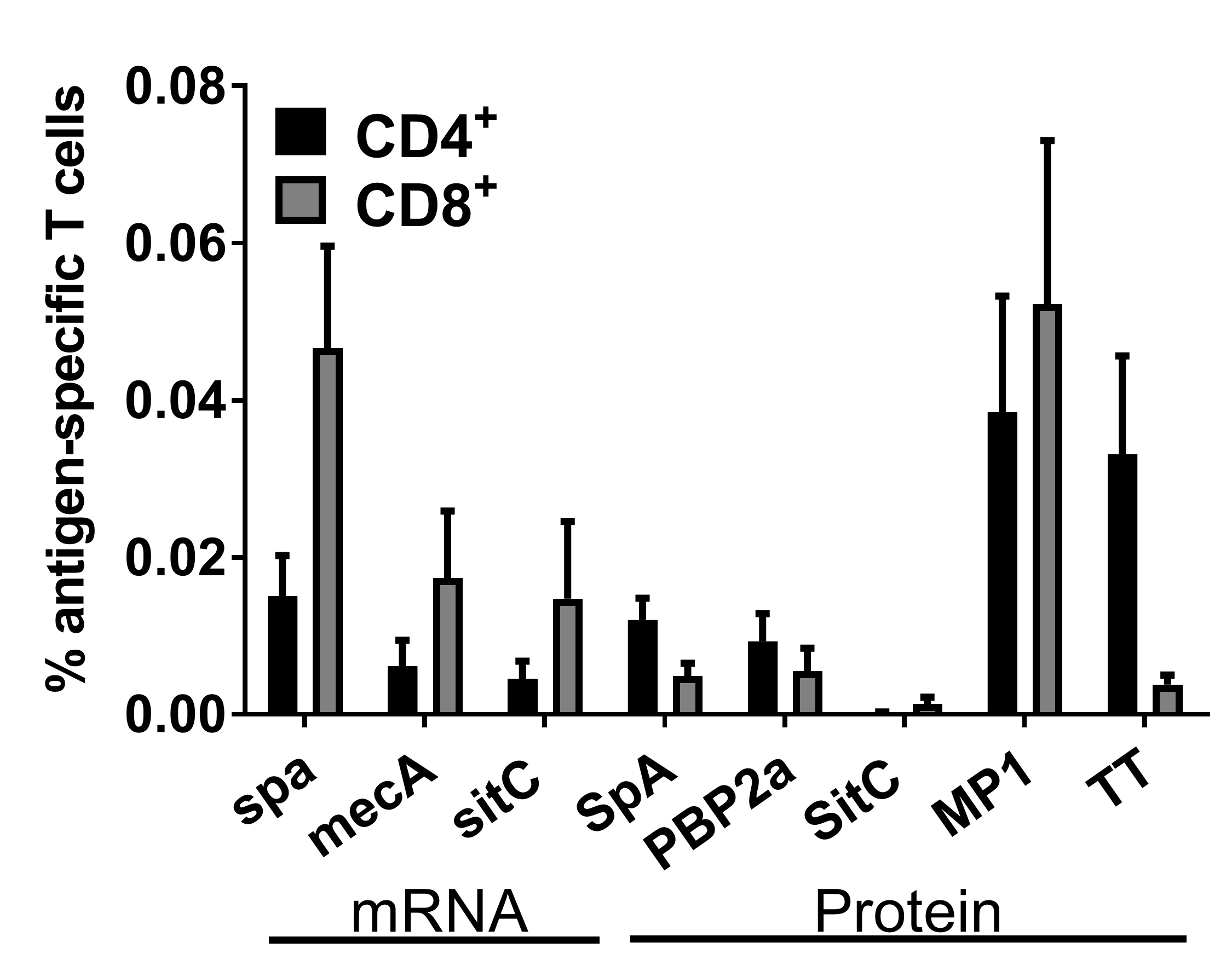

Supplement: S6 Fig — Based on the ELISPOT results (see Fig 1) the percentage of IFNγ secreting T cells was estimated by the number of spots detected by ELISPOT. For calculation of the cells triggered by mRNA stimulation, the background NC was subtracted, for the cells activated by proteins, the unstimulated control was subtracted. Results are displayed as mean ± SEM of n = 8 donors. (TIF) [file ppat.1006387.s006.tif]

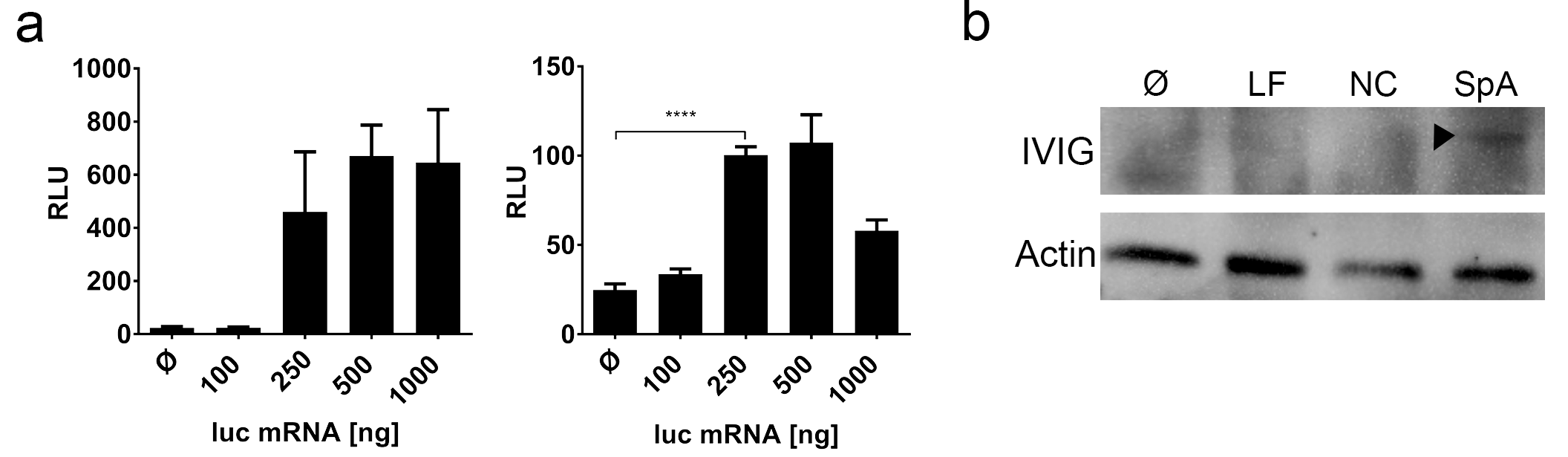

Supplement: S7 Fig — (a) HEK293 cells (left) and MoDC (right) were transfected with the indicated amounts of luc (luciferase) mRNA. Luminescence activity was measured with a luminescence plate reader in n = 2–3 independent experiments. The results obtained from triplicates are shown as mean values ± SEM. p****< 0.0001 (paired t-test) (b) Translation of ivT spa mRNA was analyzed by Western blot from lysates of transfected HEK293 cells. Data are representative out of two independent experiments. (TIF) [file ppat.1006387.s007.tif]

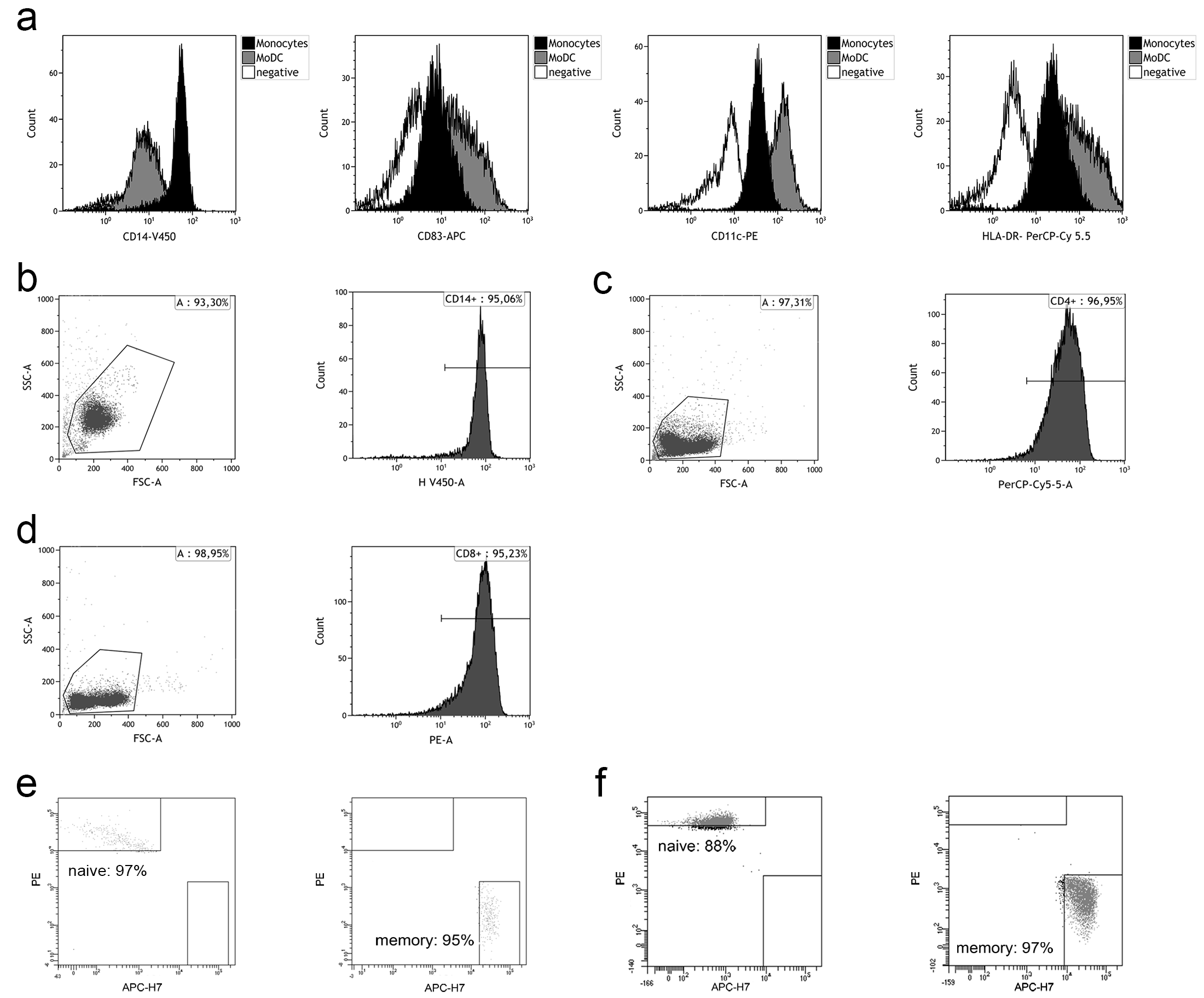

Supplement: S8 Fig — (a) Following differentiation of CD14+ monocytes, MoDC were generated for 6 days via IL-4 and GM-CSF. After 6 days, cells were harvested and MoDC phenotype was confirmed by flow cytometry using anti-CD14-V450, anti-CD83-APC, anti-CD11c-PE and anti-HLA-DR-PerCP-Cy5.5. Histograms of monocytes, corresponding MoDC and unstained negative control are shown of one representative donor out of at least six independent experiments. Purity of (b) CD14+ monocytes (c) CD4+ T cells, (d) CD8+ T cells after AutoMACS isolation was determined by flow cytometry using anti CD14-V450, anti-CD4-PerCP-Cy-5.5 and anti-CD8-PE, respectively. Purity of purified naïve and memory (e) CD4+ and (f) CD8+ T cells was confirmed by flow cytometry with anti-CD45RO-APC-H7 and anti-CD45RA-PE. Results are shown as histograms or dot plots of one representative donor. (TIF) [file ppat.1006387.s008.tif]

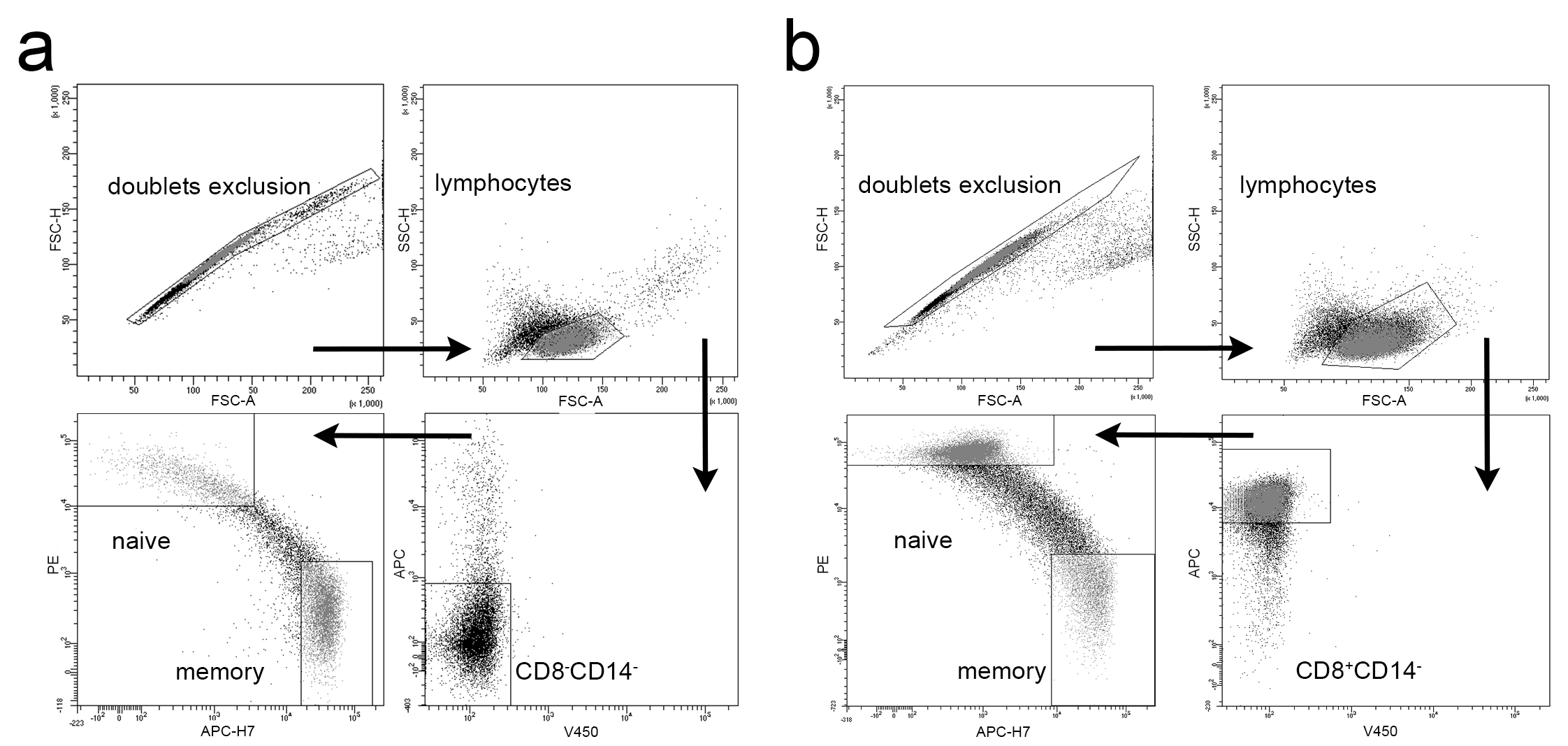

Supplement: S9 Fig — Following isolation of (a) CD4+ or (b) CD8+ cells via magnetic beads from PBMC, T cells were labeled with anti-CD45RA-PE, anti-CD45RO-APC-H7, anti-CD14-V450 and anti-CD8-APC antibodies and purified by BD FACSAria Fusion. First, doublets were excluded and cells gated for viable lymphocytes. Following exclusion CD14+ cells, naïve (CD45RA+CD45RO-) and memory (CD45RA-CD45RO+) T cells were sorted. Analysis was carried out with the BD FACS Diva software version 8.0.1. Data are representative out of at least 3 independent experiments. (TIF) [file ppat.1006387.s009.tif]
